# The generalized Hausman test for detecting non‐normality in the latent variable distribution of the two‐parameter IRT model

**DOI:** 10.1111/bmsp.12379

**Published:** 2024-12-26

**Authors:** Lucia Guastadisegni, Silvia Cagnone, Irini Moustaki, Vassilis Vasdekis

**Affiliations:** ^1^ University of Bologna Bologna Italy; ^2^ London School of Economics and Political Science London UK; ^3^ Athens University of Economics and Business Athens Greece

**Keywords:** correlated binary data, misspecification test, semi‐non‐parametric‐IRT model

## Abstract

This paper introduces the generalized Hausman test as a novel method for detecting the non‐normality of the latent variable distribution of the unidimensional latent trait model for binary data. The test utilizes the pairwise maximum likelihood estimator for the parameters of the latent trait model, which assumes normality of the latent variable, and the maximum likelihood estimator obtained under a semi‐non‐parametric framework, allowing for a more flexible distribution of the latent variable. The performance of the generalized Hausman test is evaluated through a simulation study and compared with other test statistics available in the literature for testing latent variable distribution fit and an overall goodness‐of‐fit test statistic. Additionally, three information criteria are used to select the best‐fitted model. The simulation results show that the generalized Hausman test outperforms the other tests under most conditions. However, the results obtained from the information criteria are somewhat contradictory under certain conditions, suggesting a need for further investigation and interpretation. The proposed test statistics are used in three datasets.

## 1 INTRODUCTION

This paper proposes a generalized Hausman‐type test for detecting deviation of the latent variable distribution from normality in the unidimensional latent trait model, also known as the two‐parameter model (2PL) in item response theory (IRT) (see e.g. van der Linden & Hambleton, 2013). The paper focuses on a mixture of normals and skew‐normal latent distributions.

Latent variable or trait models are used to study theoretical constructs, such as abilities, attitudes, quality of life or business confidence, which cannot be directly observed and measured. Latent variables are here continuous variables measured through correlated categorical indicators known as manifest variables or items (Bartholomew et al., 2011). This paper considers the two‐parameter latent trait model for binary manifest variables (2PL).

One of the standard assumptions of IRT models is that the latent variable(s) follows a normal distribution. However, assuming normality of the latent variable(s) when the actual distribution has a different shape can lead to biased parameter estimates, especially with binary outcomes (Ma & Genton, 2010). Furthermore, assuming an incorrect distribution of the latent variable can lead to erroneous conclusions when conducting hypothesis testing (Guastadisegni et al., 2022).

The need to accommodate more flexible latent variable distributions has been recognized in the literature. Montanari and Viroli (2010) introduced a skew‐normal latent variable in the classical factor analysis model, while Cagnone and Viroli (2012) presented a latent trait model where the factors are distributed as a finite mixture of multivariate Gaussians. Within the generalized linear latent variable model (GLLVM) framework (see e.g. Bartholomew et al., 2011; Skrondal & Rabe‐Hesketh, 2004), Ma and Genton (2010) proposed a semi‐parametric method, consistent for various types of manifest variables under different distributions of the latent variables, and Irincheeva et al. (2012) considered the semi‐non‐parametric (SNP) approach, introduced by Gallant and Nychka (1987). This approach allows for more flexible smooth densities of the latent variables. The SNP method has also been used in the unidimensional 2PL model by Woods and Lin (2009) and in the multidimensional 2PL model by Monroe (2014). For the 2PL model, Bock and Aitkin (1981) modelled the density of the latent variable using an empirical histogram instead of assuming a specific parametric form. They estimated the density together with the item parameters within an expectation‐maximization (E‐M) algorithm. The density of the latent variable was estimated directly at each quadrature point, rather than being derived from a predefined normal probability distribution. Woods and Thissen (2006) proposed a non‐parametric estimation of the latent variable while maintaining the logistic item response function (IRF). They modelled the latent variable through a Ramsey curve, which is a spline‐based density (Ramsay, 2000).

In the majority of cases, information criteria, such as the Akaike information criterion (AIC) (Akaike, 1974) or the Bayesian information criterion (BIC) (Schwarz, 1978), are used to choose between a model where the latent variables are normally distributed and a model where the latent variables have a more complex shape (Woods & Lin, 2009; Irincheeva et al., 2012; Monroe, 2014). With continuous manifest variables, Ma and Genton (2010) perform the Kolmogorov–Smirnov test on the continuous responses' distribution to evaluate the latent variable's normality.

When dealing with categorical responses, Li and Cai (2018) suggested employing summed score likelihood‐based statistics to test the fit of the latent variable distribution. They proposed two versions of the test: an unadjusted version, ${\bar{X}}^{2}$, and a moment‐adjusted version (Satorra & Saris, 1985). Similarly, Monroe (2021) introduced an alternative test statistic, denoted here by R(z), for detecting non‐normality in the latent variable distribution across the range of z. It is based on generalized residuals (Haberman & Sinharay, 2013), which compare the specified latent variable distribution to the sample average of latent variable posterior distributions, asymptotically distributed as standard normal. The paper also proposed a version to test the hypothesis that the distribution of the latent variable as a whole is correctly specified in the model denoted here as $R_{B}$. A Bonferroni correction was applied to the critical value. The statistics proposed by Li and Cai (2018) and Monroe (2021) demonstrate high power when the latent variable is skewed or platykurtic and are insensitive to multidimensionality.

Hausman (1978) proposed a specification test to detect the failure of the orthogonality assumption in regression analysis. Due to its simplicity, the Hausman test can be applied in various contexts to detect different types of model misspecification. This test compares two different estimators that are consistent when the model is correctly specified, and one is also efficient. In the presence of model misspecification, only the inefficient estimator is consistent. The efficiency assumption simplifies the covariance matrix computation of the difference between the two estimators. However, this matrix can fail to be positive definite under model misspecification or in small sample sizes. Moreover, neither of the two estimators is considered fully efficient in some cases.

The generalized version of the Hausman (GH) test, proposed by White (1982), is a more flexible and robust alternative to the original Hausman test. Indeed, the generalized version allows both estimators to be inefficient and obtained from two different models. Moreover, the covariance matrix of the difference between the two estimators is robust and always positive definite.

In the IRT context, as far as we know, the Hausman test has been used only by Ranger and Much (2020) to detect misspecification of the item characteristic functions and local dependencies among items. They highlight that this test performs well regarding Type I error rates for large sample sizes and power under most conditions. In generalized linear mixed models (GLMM) for clustered data, a robust version of the Hausman test, similar to the one by White (1982), has been proposed by Bartolucci et al. (2017) when a discrete distribution for the random effects is assumed. The test can also be used to detect the possible correlation between random effects and cluster‐specific covariates and detect endogeneity.

This work aims to extend the GH test to detect the non‐normality of the latent variable distribution in a unidimensional latent trait model for binary data. The estimators resulting from two different models are considered to build the test. The first model is a 2PL unidimensional IRT model that assumes the normality of the latent variable. In contrast, the second model is the unidimensional SNP‐IRT model, which assumes a more flexible distribution for the latent variable. The 2PL model is estimated using a pairwise maximum likelihood (PL) method (Katsikatsou et al., 2012), which is a composite likelihood method that uses information from bivariate‐order margins (Lindsay, 1988; Varin, 2008). The SNP‐IRT model is estimated using a maximum likelihood (ML) method. The choice of these estimators and models is motivated by the following reasons. First, both estimators are consistent when the latent variable is normally distributed. Moreover, the ML estimator for the SNP‐IRT model is also consistent under different distribution assumptions of the latent variable (Gallant & Tauchen, 1989; Irincheeva et al., 2012). These conditions on the consistency of the parameter estimators are required to correctly apply the GH test (White, 1982). Second, the PL and ML estimators yield different variances for the estimated parameters. This implies that under the normality of the latent variable distribution, the covariance matrix of the difference between the two estimators involved in the computation of the GH test differs from zero. The non‐zero covariance matrix avoids numerical instability in the calculation of the test.

The theoretical aspects of the models, estimators and matrices involved in the computation of the GH test are described in the following sections. Moreover, we carry out an extensive simulation study to evaluate the performance of the GH test in terms of both Type I error rates and empirical power. For the latter, we consider both a mixture of normals and skew‐normal distributions for the latent variable, with varying degrees of departure from the normal distribution. Additionally, we evaluate the asymptotic behaviour of the test in terms of both Type I error rates and power, using a very large sample size.

Furthermore, the performance of the GH test is compared with three available test statistics. Namely, the $M_{2}$ is a statistic based on standardized univariate and bivariate residuals unaffected by sparseness (Maydeu‐Olivares & Joe, 2005). The likelihood ratio (LR) test for nested models and the ${\bar{X}}^{2}$ statistic proposed by Li and Cai (2018). The latter is available in FlexMIRT (Cai, 2017). We also study the performance of three model selection information criteria. Three applications to real data are also presented.

This article is organized as follows. In Section 2, we describe the 2PL model and SNP‐IRT models for binary data and the PL and ML estimation, respectively. Section 3 presents the GH test to detect the non‐normality of the latent variable distribution. In Section 4, we review the $M_{2}$, the LR, the ${\bar{X}}^{2}$ and the $R_{B}$ test statistics, and in Section 5, the information criteria. Section 6 presents a Monte Carlo simulation study, and Section 7 presents the results from three real data analyses. Finally, in Section 8, some concluding remarks are presented and discussed.

## 2 THE 2PL AND SNP‐IRT MODEL FOR BINARY DATA

Consider n respondents answering p binary items. Let $y_{ij}\in \{0,1\}$ for i=1,…,n and j=1,…,p be a binary random variable recording individual i's response to item j. We assume that the items measure a unidimensional construct modelled by a latent variable, z. The density function of this variable is expressed as h(z).

According to the two‐parameter model (2PL), the probability of a positive response for $y_{ij}$ is modelled using a logistic model (measurement model) given by

(1) $$P(y_{ij}=1|z_{i})=\pi_{ij}(z_{i})=\frac{\exp(\alpha_{0j}+\alpha_{1j}z_{i})}{1+\exp(\alpha_{0j}+\alpha_{1j}z_{i})},\;j=1,\text{…},p$$

where $\alpha_{0j}$ is the item intercept and $\alpha_{1j}$ the item slope (factor loading) and h(z)=ϕ(z), where ϕ(z) is the standard normal density.

An extension of the 2PL model is given by the SNP‐IRT model that assumes the same response probability as (1) but a SNP parametrization of the latent variable as follows:

(2) $$h(z_{i})=P_{L}^{2}(z_{i})\phi (z_{i})\;\text{and}\;P_{L}(z_{i})=\sum_{0\leq l\leq L}a_{i}z_{i}^{l},$$

where $a_{0},\text{…},a_{L}$ are the real coefficients of the polynomial $P_{L}(z_{i})$ and L is the polynomial degree.

For h(z) to be a density, the coefficients $a_{0},\text{…},a_{L}$ of $P_{L}(z)$ should be chosen such that ∫h(z)dz=1.

We follow the same parameterization as Woods and Lin (2009) and Irincheeva et al. (2012) to obtain a unique representation of the polynomial coefficients in Equation (2). The details of this parameterization can be found in Irincheeva et al. (2012) and are reported in the Data S1: Section S1.1.

Here, we consider *L* = 0, 1 and 2. When L=0, the distribution of the latent variable in (2) reduces to the standard normal one. When L=1, $P_{L}(z)=a_{0}+a_{1}z$, where $a_{0}=\sin\varphi_{1}$, $a_{1}=\cos\varphi_{1}$, with $-\pi /2<\varphi_{1}\leq \pi /2$. The SNP parametrization with L=1 includes unimodal and bimodal distributions.

**FIGURE 1:**
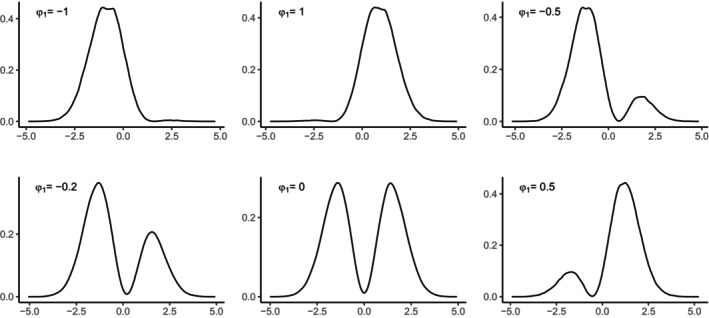
SNP densities of z when L=1, for different values of the $\varphi_{1}$ parameter.

Figure 1 displays the SNP densities of z when L=1, for different values of the $\varphi_{1}$ parameter.

When $\varphi_{1}$ is negative and close to −1, the distribution is slightly right‐skewed, whereas when it is close to 1, it is slightly left‐skewed. When the values of $\varphi_{1}$ are between −1 and 1, the distributions are bimodal. Even if not reported in the graph, when $\varphi_{1}=\frac{\pi }{2}$, the SNP distribution reduces to the standard normal.

**FIGURE 2:**
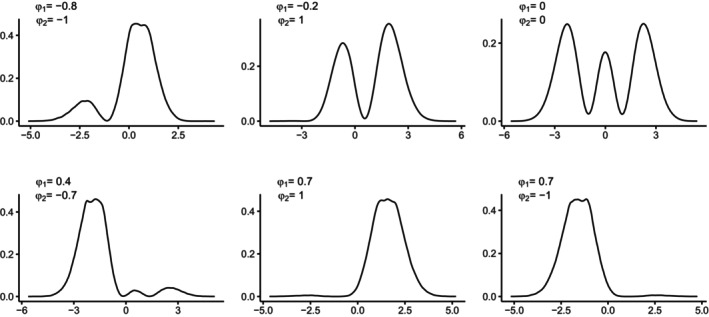
SNP densities of z when L=2, for different values of the $\varphi_{1}$ and $\varphi_{2}$ parameters.

When L=2, $P_{L}(z)=a_{0}+a_{1}z+a_{2}z^{2}$, $a_{0}=\sin\varphi_{1}-\frac{1}{\sqrt{2}}\cos\varphi_{1}\cos\varphi_{2}$, $a_{1}=\cos\varphi_{1}\sin\varphi_{2}$ and $a_{2}=\frac{1}{\sqrt{2}}\cos\varphi_{1}\cos\varphi_{2}$, with $-\pi /2<\varphi_{l}\leq \pi /2$, l=1,2. The SNP parametrization with L=2 is more flexible than L=1 and encompasses unimodal, multimodal (including up to three modes) and skewed distributions. Figure 2 displays the SNP densities of z when L=2, for different values of the $\varphi_{1}$ and $\varphi_{2}$ parameters.

When the value of $\varphi_{1}$ is negative, and the value of $\varphi_{2}$ is close to −1 or 1, the distributions are bimodal. When both parameters are close to 0, the distributions are trimodal. When the value of $\varphi_{1}$ is positive and >0.5, and the value of $\varphi_{2}$ is close to −1 or 1, the distributions are highly right‐skewed and left‐skewed, respectively. Even though it is not reported in Figure 2, when $\varphi_{2}=\frac{\pi }{2}$, the SNP densities reduce to those observed when L=1. Moreover, when both parameters are set to $\frac{\pi }{2}$, the SNP densities return to the standard normal.

We indicate with ${\text{SNP}}_{2}$ the 2PL model for L=2, with ${\text{SNP}}_{1}$ the 2PL model for L=1 and with ${\text{SNP}}_{0}$ the 2PL model for L=0.

### 2.1 PL estimation for the ${\text{SNP}}_{0}$ model

To implement the GH test, the parameters of the ${\text{SNP}}_{0}$ model are estimated with the PL. The pairwise log‐likelihood, based on the bivariate marginal densities $f(y_{ij},y_{ik},\theta )$, j,k=1,..p and k>j, is

(3) $$\begin{array}{ll} & pl_{{\text{SNP}}_{0}}(y,\theta )=\overset{n}{\underset{i=1}{\sum }}\overset{p}{\underset{j=1}{\sum }}\underset{k>j}{\sum }\ln f(y_{ij},y_{ik},\theta )=\\ & =\overset{n}{\underset{i=1}{\sum }}\overset{p}{\underset{j=1}{\sum }}\underset{k>j}{\sum }\ln\int [\pi_{ij}{\left(z_{i}\right)}^{y_{ij}}{\left(1-\pi_{ij}(z_{i})\right)}^{1-y_{ij}}][\pi_{ik}{\left(z_{i}\right)}^{y_{ik}}{\left(1-\pi_{ik}(z_{i})\right)}^{1-y_{ik}}]\phi (z_{i})dz_{i}.\; \end{array}$$

$pl_{{\text{SNP}}_{0}}(y,\theta )$ is maximized with respect to θ, where θ includes the item intercepts and slopes. Under correct model specification, the maximum PL estimator $\tilde{\theta }$ converges in probability to the true parameter vector $\theta_{0}^{\prime }=(\alpha_{00}^{\prime },\alpha_{01}^{\prime })$ and

(4) $$\tilde{\theta }\overset{p}{\to }N(\theta_{0},A^{-1}(\theta_{0})B(\theta_{0})A^{-1}(\theta_{0})),$$

where $A(\theta )=E_{y}[-\frac{\partial^{2}pl_{{\text{SNP}}_{0}}(y,\theta )}{\partial \theta \partial \theta^{\prime }}]$, $B=var[\frac{\partial pl_{{\text{SNP}}_{0}}(y,\theta )}{\partial \theta }]$ and A(θ)≠B(θ) (Lindsay, 1988, Varin, 2008). These matrices can be estimated by their observed versions evaluated at $\tilde{\theta }$ as

(5) $$Â(\tilde{\theta })={\left.\overset{n}{\underset{i=1}{\sum }}\frac{\partial^{2}pl_{{\text{SNP}}_{0}}(y_{i},\theta )}{\partial \theta \partial \theta^{\prime }}\right|}_{\theta =\tilde{\theta }}$$

and

(6) $$\hat{B}(\tilde{\theta })={\left.\overset{n}{\underset{i=1}{\sum }}\frac{\partial pl_{{\text{SNP}}_{0}}(y_{i},\theta )}{\partial \theta }\frac{\partial pl_{{\text{SNP}}_{0}}(y_{i},\theta )}{\partial \theta^{\prime }}\right|}_{\theta =\tilde{\theta }}.$$

### 2.2 ML estimator for the SNP_L_ model

The parameter vector $\theta^{(1)\prime }=(\alpha_{0}^{\prime },\alpha_{1}^{\prime },\varphi \prime )=(\theta \prime ,\varphi \prime )$ of the ${\text{SNP}}_{L}$ model, where L>0, is estimated using the ML method. The log‐likelihood of the data is 
(7)

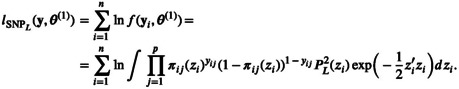

The integral in $l_{{\text{SNP}}_{L}}(y,\theta^{\left(1\right)})$ is approximated by using the Gauss–Hermite quadrature, as in Woods and Lin (2009). The degree of the polynomial L is fixed and is not estimated by ML. The log‐likelihood function is maximized with respect to the unknown vector of parameter $\theta^{\left(1\right)}$ as follows:

(8) $$({\dot{\alpha }}_{0}^{\prime },{\dot{\alpha }}_{1}^{\prime },{\hat{\varphi }}^{\prime })=argmax_{\theta^{\left(1\right)}}l_{{\text{SNP}}_{L}}(y,\theta^{\left(1\right)}).$$

When the model is correctly specified, this is the full ML method. Under model misspecification, the method becomes a quasi‐ML method (White, 1982). The two methods are computationally the same, but the estimators exhibit different theoretical properties, as will be clarified later in this section.

Regarding the identifiability of the model parameters with respect to the arbitrariness of the location and scale of the latent variable, we show in Section S1.4 of Data S1 that model parameters for the ${\text{SNP}}_{1}$ model are locally identifiable without having to fix the location and scale of the latent variable or the elements of the loading matrix to specified values. This is not the case for most of the latent variable models with a parametric distribution for the latent variable. In the same way, for larger SNP models, one can show that high‐order moments of the observed variable contribute to the estimation of model parameters, thus providing at least locally identified model parameters. Irincheeva et al. (2012) also discusses the identifiability due to rotational indeterminacy.

After maximizing the log‐likelihood in (7), the algorithm converges to $\left({\dot{\alpha }}_{0}^{\prime },{\dot{\alpha }}_{1}^{\prime },{\hat{\varphi }}^{\prime }\right)$ and the latent variable z has a density $h(z|\hat{\varphi },L)$ with an estimated mean $\tilde{E}(Z)$ and variance $\tilde{V}(Z)$ (see Section S1.2 of Data S1 for details). The estimators ${\dot{\alpha }}_{0}$ and ${\dot{\alpha }}_{1}$ are, in effect, calibrated with respect to the $h(z|\hat{\varphi },L)$ distribution. However, the estimates can be rescaled to compare with those of the 2PL with a standard normal latent variable distribution, as discussed in Section S1.3 of Data S1. The rescaled parameters are denoted as ${\hat{\theta }}^{(1)\prime }=({\hat{\alpha }}_{0}^{\prime },{\hat{\alpha }}_{1}^{\prime },{\hat{\varphi }}^{\prime })$.

The SNP densities can approximate various distributions, including a mixture of normals and skew distributions. However, as in almost all cases the SNP densities approximate the true latent variable distribution but do not exactly coincide with it, if the regularity conditions A2–A6 of White (1982) are satisfied, the obtained estimator is a Quasi‐ML estimator

(9) $${\hat{\theta }}^{\left(1\right)}\;\overset{p}{\to }N\;\left(\theta_{0}^{\left(1\right)},A^{-1}\;\left(\theta_{0}^{\left(1\right)}\right)\;B\;\left(\theta_{0}^{\left(1\right)}\right)\;A^{-1}\;\left(\theta_{0}^{\left(1\right)}\right)\right),$$

where $\theta_{0}^{(1)\prime }=(\alpha_{00}^{\prime },\alpha_{01}^{\prime },\varphi_{*}^{\prime })=(\theta_{0}^{\prime },\varphi_{*}^{\prime })$. $\varphi_{*}$ is the value of φ that minimizes the Kullback–Leibler information criterion (White, 1982; Gallant & Tauchen, 1989; Irincheeva et al., 2012). If the true latent variable follows exactly an SNP_*L*_ density, including the normal as a sub‐case, that is, when $\varphi_{l}=\frac{\pi }{2}$, the vector $\varphi_{*}$ coincides with the true parameter value $\varphi_{0}$, and the quasi‐ML method reduces to the full ML method. A(θ) and B(θ) are the expected Hessian and cross‐product matrices, respectively. Their observed versions can be computed with the Delta method (Cramér, 1946) and are defined similar to (5) and (6), where $pl_{{\text{SNP}}_{0}}(y_{i},\theta )$ is replaced by $l_{{\text{SNP}}_{L}}(y_{i},\theta^{\left(1\right)})$.

## 3 THE GENERALIZED HAUSMAN TEST

In this section, we present the GH test, derived by White (1982), applied here to detect the non‐normality of the latent variable using the SNP‐IRT model.

As in the previous sections, let us denote by θ the sub‐vector of $\theta^{(1)\prime }=(\alpha_{0}^{\prime },\alpha_{1}^{\prime },\varphi \prime )$ that includes the item intercepts $\alpha_{0}$ and slopes $\alpha_{1}$. θ has dimension 2p×1, where p is the number of items.

For the 2PL IRT model (${\text{SNP}}_{0}$), consider the maximum PL estimator ${\tilde{\theta }}_{{\text{SNP}}_{0}}$.

Consider the ML estimator ${\hat{\theta }}_{{\text{SNP}}_{L}}^{(1)\prime }=({\hat{\theta }}_{{\text{SNP}}_{L}}^{\prime },{\hat{\varphi }}^{\prime })$ of a SNP‐IRT model with L>0, where the sub‐vector of parameter $\hat{\varphi }$ has dimension L×1 and so ${\hat{\theta }}_{{\text{SNP}}_{L}}^{\left(1\right)}$ has dimension (2p+L)×1.

Following White (1982), under normality of the latent variable

(10) $$\sqrt{n}\;\left({\hat{\theta }}_{{\mathrm{SNP}}_{L}}-{\tilde{\theta }}_{{\mathrm{SNP}}_{0}}\right)\;\overset{d}{\to }\;N\left(0,S\left(\theta_{0},\theta_{0}^{\left(1\right)}\right)\right).$$

An estimator of $S(\theta_{0},{\theta_{0}}^{\left(1\right)})$ is given by

(11) $$\begin{array}{ll} & Ŝ({\tilde{\theta }}_{{\text{SNP}}_{0}},{\hat{\theta }}_{{\text{SNP}}_{L}}^{\left(1\right)})={Â}^{\theta \varphi }{\left({\hat{\theta }}_{{\text{SNP}}_{L}}^{\left(1\right)}\right)}^{-1}\hat{B}({\hat{\theta }}_{{\text{SNP}}_{L}}^{\left(1\right)}){Â}^{\theta \varphi }{\left({\hat{\theta }}_{{\text{SNP}}_{L}}^{\left(1\right)}\right)}^{-1^{\prime }}+Â{\left({\tilde{\theta }}_{{\text{SNP}}_{0}}\right)}^{-1}\hat{B}({\tilde{\theta }}_{{\text{SNP}}_{0}})Â{\left({\tilde{\theta }}_{{\text{SNP}}_{0}}\right)}^{-1^{\prime }}-\\ & \;-{Â}^{\theta \varphi }{\left({\hat{\theta }}_{{\text{SNP}}_{L}}^{\left(1\right)}\right)}^{-1}\hat{R}{\left({\tilde{\theta }}_{{\text{SNP}}_{0}},{\hat{\theta }}_{{\text{SNP}}_{L}}^{\left(1\right)}\right)}^{\prime }Â{\left({\tilde{\theta }}_{{\text{SNP}}_{0}}\right)}^{-1^{\prime }}-Â{\left({\tilde{\theta }}_{{\text{SNP}}_{0}}\right)}^{-1}\hat{R}({\tilde{\theta }}_{{\text{SNP}}_{0}},{\hat{\theta }}_{{\text{SNP}}_{L}}^{\left(1\right)}){Â}^{\theta \varphi }{\left({\hat{\theta }}_{{\text{SNP}}_{L}}^{\left(1\right)}\right)}^{-1^{\prime }},\; \end{array}$$

where the matrices $Â({\tilde{\theta }}_{{\text{SNP}}_{0}})$ and $\hat{B}({\tilde{\theta }}_{{\text{SNP}}_{0}})$, defined in Equations (5) and (6), have dimension 2p×2p and are evaluated at ${\tilde{\theta }}_{{\text{SNP}}_{0}}$. $Â({\hat{\theta }}_{{\text{SNP}}_{L}}^{\left(1\right)})$ and $\hat{B}({\hat{\theta }}_{{\text{SNP}}_{L}}^{\left(1\right)})$ are the observed Hessian and cross‐product matrix of dimension (2p+L)×(2p+L) for the ${\text{SNP}}_{L}$ model, evaluated at ${\hat{\theta }}_{{\text{SNP}}_{L}}^{\left(1\right)}$. The matrix ${Â}^{\theta \varphi }{\left({\hat{\theta }}_{{\text{SNP}}_{L}}^{\left(1\right)}\right)}^{-1}$ is obtained by deleting the last L rows from the matrix $Â{\left({\hat{\theta }}_{{\text{SNP}}_{L}}^{\left(1\right)}\right)}^{-1}$ and has dimension 2p×(2p+L). The matrix $\hat{R}({\tilde{\theta }}_{{\text{SNP}}_{0}},{\hat{\theta }}_{{\text{SNP}}_{L}}^{\left(1\right)})$ has dimension 2p×(2p+L) and can be computed as

(12) $$\hat{R}(\theta_{{\text{SNP}}_{0}},\theta_{{\text{SNP}}_{L}}^{\left(1\right)})=\overset{n}{\underset{i=1}{\sum }}\frac{\partial pl_{{\text{SNP}}_{0}}(y_{i},\theta )}{\partial \theta }\frac{\partial l_{{\text{SNP}}_{L}}(y_{i},\theta^{\left(1\right)})}{\partial \theta^{{\left(1\right)}^{\prime }}},$$

where $pl_{{\text{SNP}}_{0}}(y_{i},\theta )$ is the pairwise log‐likelihood for the individual i under the model ${\text{SNP}}_{0}$ and $l_{{\text{SNP}}_{L}}(y_{i},\theta^{\left(1\right)})$ is the log‐likelihood for the individual i under the model ${\text{SNP}}_{L}$. The matrix in (12) is evaluated at $\left({\tilde{\theta }}_{{\text{SNP}}_{0}},{\hat{\theta }}_{{\text{SNP}}_{L}}^{\left(1\right)}\right)$. We choose the maximum PL and the ML estimator for the two models to avoid that, under correct model specification, ${\tilde{\theta }}_{{\text{SNP}}_{0}}$ and ${\hat{\theta }}_{{\text{SNP}}_{L}}$ converge to the same covariance matrix, producing a $Ŝ({\tilde{\theta }}_{{\text{SNP}}_{0}},{\hat{\theta }}_{{\text{SNP}}_{L}}^{\left(1\right)})$ matrix in (11) with all entries close to 0.

Given the theoretical result in (10), the GH test is given by

(13) $$\text{GH}={\left({\hat{\theta }}_{{\text{SNP}}_{L}}-{\tilde{\theta }}_{{\text{SNP}}_{0}}\right)}^{\prime }Ŝ{\left({\tilde{\theta }}_{{\text{SNP}}_{0}},{\hat{\theta }}_{{\text{SNP}}_{L}}^{\left(1\right)}\right)}^{-1}({\hat{\theta }}_{{\text{SNP}}_{L}}-{\tilde{\theta }}_{{\text{SNP}}_{0}}).$$

Under normality of the latent variable, the GH test is asymptotically distributed as a $\chi_{2p}^{2}$, with 2p degrees of freedom, that is, the number of parameters in θ.

We consider a simpler version of (13) that does not involve the inversion of the matrix $Ŝ({\tilde{\theta }}_{{\text{SNP}}_{0}},{\hat{\theta }}_{{\text{SNP}}_{L}}^{\left(1\right)})$ giving

(14) $${\text{GH}}_{T}={\left({\hat{\theta }}_{{\text{SNP}}_{L}}-{\tilde{\theta }}_{{\text{SNP}}_{0}}\right)}^{\prime }I_{2p}^{-1}({\hat{\theta }}_{{\text{SNP}}_{L}}-{\tilde{\theta }}_{{\text{SNP}}_{0}}),$$

where $I_{2p}$ can be omitted from the above formula.

Following Yuan and Bentler (2010)

(15) $${\text{GH}}_{T}=\overset{d}{\underset{l=1}{\sum }}\lambda_{l}\delta_{l}^{2},\delta_{l}∼N(0,1),$$

where d is the rank of $S(\theta_{0},{\theta_{0}}^{\left(1\right)})$ and $\lambda_{1},\text{…},\lambda_{d}$ are its non‐zero eigenvalues.

It is possible to approximate the distribution of ${\text{GH}}_{T}$ using the moment matching method (Welch, 1938; Yuan & Bentler, 2010) as follows:

(16) $${\text{GH}}_{T}∼a\chi_{b}^{2}.$$

The quantities a and b are defined as

(17) $$a=\frac{\sum_{l=1}^{d}\lambda_{l}^{2}}{\sum_{l=1}^{d}\lambda_{l}}$$

and

(18) $$b=\frac{{\left(\sum_{l=1}^{d}\lambda_{l}\right)}^{2}}{\sum_{l=1}^{d}\lambda_{l}^{2}}.$$

As $S(\theta_{0},{\theta_{0}}^{\left(1\right)})$ can be consistently estimated by $Ŝ({\tilde{\theta }}_{{\text{SNP}}_{0}},{\hat{\theta }}_{{\text{SNP}}_{L}}^{\left(1\right)})$ defined in (11), a and b can be consistently estimated substituting ${\hat{\lambda }}_{1},\text{…},{\hat{\lambda }}_{d}$ in (17) and (18), where d is rank of Ŝ and ${\hat{\lambda }}_{1},\text{…},{\hat{\lambda }}_{d}$ are its non‐zero eigenvalues. The approximation in (16) matches the first two moments of ${\text{GH}}_{T}$ with those of $a\chi_{b}^{2}$ (16).

Similar to the statistics derived by Monroe (2021) and Li and Cai (2018), the ${\text{GH}}_{T}$ test can be sensitive to the misspecification of either the latent variable distribution or the IRF. For this reason, correct IRFs for the items are assumed when testing the normality of the latent variable.

To study the sensitivity of the proposed ${\text{GH}}_{T}$ test to misspecification of the IRF, we run a small‐scale simulation and provide the results in Section S2.3 of Data S1.

## 4 AVAILABLE TEST STATISTICS FOR TESTING LATENT VARIABLE DISTRIBUTION FIT AND THE OVERALL FIT

In addition to the proposed test statistic, we review three test statistics for testing latent variable distribution fit and one for the overall goodness‐of‐fit of the model considered each time.

An LR test statistic for nested models can be used since the ${\text{SNP}}_{L}$ and ${\text{SNP}}_{0}$ models are nested (Wilks, 1938). The ${\text{SNP}}_{L}$ model, when $\varphi_{1}=\text{⋯}=\varphi_{L}=\frac{\pi }{2}$, reduces to the ${\text{SNP}}_{0}$ model (Irincheeva et al., 2012). For the computation of the LR test, the ${\text{SNP}}_{0}$ model needs to be estimated using ML instead of pairwise likelihood to obtain a comparable value of the log‐likelihood function with that of the ${\text{SNP}}_{L}$ model.

Let us denote by $\varphi^{\prime }=(\varphi_{1},\text{⋯}\;,\varphi_{L})$ and by ${\text{c}}^{\prime }=(\frac{\pi }{2},\text{⋯}\;,\frac{\pi }{2})$. The null and alternative hypotheses can be formulated as follows:

(19) $$H_{0}:\varphi =\text{c}\;\text{vs}\;H_{1}:\varphi \neq \text{c}.$$

The test statistic is defined as

(20) $$\text{LR}=2\{l_{{\text{SNP}}_{L}}(y,{\hat{\theta }}^{\left(1\right)})-l_{{\text{SNP}}_{0}}(y,\hat{\theta })\},$$

where $l_{{\text{SNP}}_{L}}(y,{\hat{\theta }}^{\left(1\right)})$ and $l_{{\text{SNP}}_{0}}(y,\hat{\theta })$ are the log‐likelihood functions of the ${\text{SNP}}_{L}$ and ${\text{SNP}}_{0}$ models, respectively, evaluated at their maximum values. Under $H_{0}$, the LR test is asymptotically distributed as a $\chi_{L}^{2}$, where L is the degree of the polynomial. For a fixed sample size, the number of empty cells in a frequency table increases with the number of binary items. In this case, the distribution of the LR test statistic is not well approximated by the chi‐square distribution.

Li and Cai (2018) proposed a Pearson‐type statistic based on summed scores given by:

(21) $${\bar{X}}^{2}=n\overset{S-1}{\underset{s=0}{\sum }}\frac{{\left({\bar{p}}_{s}-{\bar{\pi }}_{s}\right)}^{2}}{{\bar{\pi }}_{s}},$$

where n is the sample size, S=1+p represents the possible summed scores with p binary items, ranging from 0 to S−1. ${\bar{p}}_{s}$ and ${\bar{\pi }}_{s}$ denote the observed summed score proportions and the corresponding model‐implied summed score probabilities computed under an IRT model, respectively, for s=0,⋯,S−1.

Li and Cai (2018) conjectured that under a wide variety of conditions, the tail‐area probabilities of ${\bar{X}}^{2}$ can be well approximated by a $\chi^{2}$ distribution with S−1−2 degrees of freedom under the null hypothesis that the latent variable distribution is correctly specified in the IRT model. The authors also proposed a moment‐adjusted version of this test.

Monroe (2021) proposed a test statistic based on posterior residuals written as:

(22) $$R(z)=\frac{\bar{f}(z|\text{y},\hat{\theta })-d(z;\hat{\theta })}{s(z)},$$

where $\hat{\theta }$ denotes the ML estimates of the free parameters of the IRT model, $\bar{f}(z|\text{y},\hat{\theta })=n^{-1}\sum_{i=1}^{n}f(z|{\text{y}}_{i},\hat{\theta })$ denotes the sample average of the posterior distribution, $d(z;\hat{\theta })$ is the ML estimate of the distribution of the latent variable and s(z) is the corresponding standard error estimate. If d(z;θ) is assumed to be standard normal, then R(z) compares the posterior average, $\bar{f}(z|\text{y},\hat{\theta })$, with the standard normal distribution. A version of this test statistic for overall latent variable distribution fit, denoted by $R_{B}$, is defined as the maximum absolute of R(z) and compared to a Bonferroni‐corrected critical value.

We also review one more test statistic for the overall fit of the ${\text{SNP}}_{0}$ model proposed by Maydeu‐Olivares and Joe (2005). Although not developed for testing latent distribution fit, it provides an overall goodness‐of‐fit test statistic that can work well under sparseness. Maydeu‐Olivares and Joe (2005) propose a family of test statistics $M_{r}$, based on the residuals up to order r. The most popular statistic is $M_{2}$, which uses the univariate and bivariate marginal information. As data sparseness increases, the empirical Type I error rates of the $M_{2}$ test remain accurate (Maydeu‐Olivares & Joe, 2005, 2006). Under the null hypothesis, we test that the ${\text{SNP}}_{0}$ model holds. The hypotheses $H_{0}$ and $H_{1}$ can be formalized as follows:

(23) $$H_{0}:\pi =\pi (\theta )\;\text{vs}\;H_{1}:\pi \neq \pi (\theta ),$$

where θ, as usual, includes the item intercepts and slopes and π(θ) indicates the response patterns probabilities.

The test statistic $M_{2}$ is (Maydeu‐Olivares & Joe, 2005):

(24) $$M_{2}=n{\hat{\text{e}}}_{2}^{\prime }{\hat{U}}_{2}{\hat{\text{e}}}_{2}.$$

The vector $\hat{\text{e}}$ includes the univariate and bivariate residuals while the matrix ${\hat{U}}_{2}$ depends on a transformation matrix and the Jacobian matrix of the cell probabilities with respect to the items intercept and slope parameter (more details can be found in (Maydeu‐Olivares & Joe, 2005, 2006). Under $H_{0}$, the statistic $M_{2}$ is asymptotically distributed as a $\chi_{m}^{2}$, with degrees of freedom $m=\frac{p(p+1)}{2}-2p$, that is the number of univariate and bivariate residuals minus the number of estimated parameters of the ${\text{SNP}}_{0}$ model.

In the simulations, we evaluate the performance of the LR, ${\bar{X}}^{2}$ and $M_{2}$ test statistics under normality and non‐normality of the latent variable distribution. However, $R_{B}$ is not available in commercial software and has therefore been reported only in the real data application in Section 7.1, where it was previously computed.

## 5 MODEL SELECTION CRITERIA

The AIC, the BIC and the Hannan–Quinn criterion (HQ) can be used to choose the degree of the polynomial L of the SNP‐IRT model (Davidian & Gallant, 1993; Woods & Lin, 2009; Irincheeva et al., 2012; Monroe, 2014).

The AIC is (Akaike, 1974):

(25) $$\text{AIC}=-2l(y,{\hat{\theta }}^{\left(1\right)})+2k,$$

where $l(y,{\hat{\theta }}^{\left(1\right)})$ is the maximum value of the log‐likelihood function of the ${\text{SNP}}_{L}$ model and k is the number of free mode parameters.

The BIC is (Schwarz, 1978):

(26) $$\text{BIC}=-2l(y,{\hat{\theta }}^{\left(1\right)})+k\ln n,$$

where n is the sample size.

The HQ is (Hannan & Quinn, 1979):

(27) $$\text{HQ}=-2l(y,{\hat{\theta }}^{\left(1\right)})+2k\ln\ln n.$$

Usually, L=1 or L=2 is enough to detect a departure from normality and approximate different latent variable distribution shapes. Selecting a higher order of the polynomial could result in overfitting (Irincheeva, 2011).

When L=0, ${\hat{\theta }}^{\left(1\right)}$ equals $\hat{\theta }$ in the aforementioned formulas.

## 6 SIMULATION STUDY

### 6.1 Design

In this section, we study the performance of the ${\text{GH}}_{T}$ test to assess the non‐normality of the latent variable distribution, and we compare its performance with the LR, ${\bar{X}}^{2}$ and $M_{2}$ test statistics. Moreover, the BIC, AIC and HQ criteria were computed for all simulation scenarios.

We have considered five scenarios (SC) corresponding to five distribution assumptions for the latent variable z in the data‐generating models. The unidimensional latent trait model is

(28) $$\begin{array}{ll} & \text{logit}(\pi_{ij})=\alpha_{0j}+\alpha_{1j}z_{i}\;i=1,\text{…},n\;j=1,2,\text{…},p. \end{array}$$

Item intercepts, $\alpha_{0j}$, have been randomly chosen from the interval [−.8; 1.12] while the item slopes, $\alpha_{1j}$, from the interval [.5; 1.5]. To study the Type I error rates of the above four test statistics, we have considered the following scenario:

A z∼N(0,1).

For the power, we have considered the following cases of two bimodal distributions and two skewed distributions:

B z∼.7N(−1,.7)+.3N(1,.8),
  where z has an overall mean of −.40 and a variance of 1.38.

C z∼.1N(−2,.25)+.9N(2,1),
  where z has an overall mean of 1.6 and a variance of 2.37.

D Z∼SN(μ=−2.5,σ=2,λ=5),
  where z has a mean of −.93 and a variance of 1.55.

E Z∼SN(μ=−2.5,σ=2,λ=10),
  where z has a mean of −.91 and a variance of 1.47.

The degree of deviation from the normal distribution is greater in scenario C than in scenario B, and similarly, the skew‐normal distribution in scenario E exhibits a greater deviation from the normal distribution than the one in scenario D.

In the simulations, two versions of the ${\text{GH}}_{T}$ test were considered. The first version, named ${\text{GH}}_{T1}$, is based on the ${\text{SNP}}_{0}$ and ${\text{SNP}}_{1}$ models. The ${\text{SNP}}_{1}$ model was chosen because it can approximate bimodal distributions well, present in scenarios B and C.

The ${\text{SNP}}_{1}$ model has been optimized in R using the ‘nlminb’ function, which maximizes directly through the analytically computed gradient and Hessian matrix. In the case of the ${\text{SNP}}_{1}$ model, the initial values for the parameters $\alpha_{0j}$ and $\alpha_{1j}$ used in the optimization process are the ML parameter estimates obtained with the ${\text{SNP}}_{0}$ model. For the $\varphi_{1}$ parameter, we have sampled 10 initial values from a sequence of values equally spaced by .1 within the domain of $\varphi_{1}$ which is the interval $\left[-\frac{\pi }{2};\frac{\pi }{2}\right]$. From the estimated ${\text{SNP}}_{1}$ models in each data replication, we selected the one corresponding to the maximum value of the quasi‐log‐likelihood function. The ${\text{GH}}_{T1}$ test has been evaluated under all scenarios to assess its performance.

In scenarios D and E, a second version of ${\text{GH}}_{T}$ called ${\text{GH}}_{T2}$ has been considered in addition to ${\text{GH}}_{T1}$. ${\text{GH}}_{T2}$ is based on the ${\text{SNP}}_{0}$ and ${\text{SNP}}_{2}$ models, suitable for capturing highly skewed cases when L=2, as shown in Figure 2. The ${\text{SNP}}_{2}$ model optimization uses the direct maximization function ‘nlminb’ in R. However, it is susceptible to the initial values chosen. To overcome this, the true parameter values have been used as initial values for the $\alpha_{0j}$ and $\alpha_{1j}$ parameters, and the initial values for the $\varphi_{1}$ and $\varphi_{2}$ parameters have been set to 0.7 and 1, respectively, corresponding to a skew‐normal case. To compute the ${\text{GH}}_{T1}$ and ${\text{GH}}_{T2}$ test statistics, the ${\text{SNP}}_{0}$ model has been estimated using the maximum PL method through the R function ‘optim’. The Hessian, cross‐product matrices and the matrix in formula (12) involved in the ${\text{GH}}_{T1}$ and ${\text{GH}}_{T2}$ tests have been computed numerically with the ‘NumDeriv’ R package.

To compute the values of the information criteria and the LR test, the ${\text{SNP}}_{0}$ model has been estimated using ML in R via the ‘optim’ function. For the $M_{2}$ test, we also used ML estimation. We estimated the 2PL model with the expectation‐maximization (E‐M) algorithm with the ‘mirt’ function in R. Then, we applied the ‘M2’ function to calculate the test statistic.

We used flexMIRT (Cai, 2017) to compute ${\bar{X}}^{2}$.

The Type I error rates and power of the ${\text{GH}}_{T1},{\text{GH}}_{T2}$, LR, ${\bar{X}}^{2}$ and $M_{2}$ have been computed using the following formula: $\hat{p}=\sum_{l=1}^{N_{v}}\frac{I(T_{l}\geq c)}{N_{v}}$. Here, $N_{v}$ represents the number of valid replications out of the total, I denotes an indicator function and $T_{l}$ is the test statistic value evaluated in the l‐th replication. The critical value c corresponds to the theoretical asymptotic value, specifically the (1−α)th percentile of the $â\chi_{\hat{b}}^{2}$ distribution for the ${\text{GH}}_{T}$ test. The values â and $\hat{b}$ are computed as in (17) and (18). For the $M_{2}$ test, the critical value is associated with the $\chi_{m}^{2}$ distribution, where m is equal to $\frac{p(p+1)}{2}-2p$. For ${\bar{X}}^{2}$, the critical value is associated with the $\chi_{S-1-2}^{2}$ distribution.

To compare the performance of two models, ${\text{SNP}}_{1}$ and ${\text{SNP}}_{0}$, we use the LR test. As ${\text{SNP}}_{1}$ has an additional parameter compared to ${\text{SNP}}_{0}$, we use c as the theoretical asymptotic critical value corresponding to the (1−α)th percentile of the $\chi_{1}^{2}$ distribution. This specific test is called ${\text{LR}}_{1}$. To compare ${\text{SNP}}_{0}$ with ${\text{SNP}}_{2}$, we use the LR test with two degrees of freedom, denoted as ${\text{LR}}_{2}$. A confidence interval (CI) of each rate $\hat{p}$ is computed as $\hat{p}\pm z_{\left(1-\frac{\alpha }{2}\right)}\sqrt{\frac{\alpha (1-\alpha )}{N_{v}}}$.

Next, the percentages of times the AIC, BIC and HQ criteria select the ${\text{SNP}}_{1}$ over ${\text{SNP}}_{0}$ have been computed as $\hat{P}=\sum_{l=1}^{N_{v}}\frac{I(IC_{{\text{SNP}}_{1}}<IC_{{\text{SNP}}_{0}})}{N_{v}}100$, where IC indicates the AIC, BIC and HQ criteria. Similarly, we computed percentages for selecting the ${\text{SNP}}_{2}$ model over the ${\text{SNP}}_{0}$ model using information criteria.

We have conducted simulations for scenarios A, B and C with the following conditions: number of items (p=10,20), sample size (n=500,1000,5000), test statistics (${\text{GH}}_{T1}$, ${\text{LR}}_{1}$, ${\bar{X}}^{2}$, $M_{2}$) and information criteria (AIC, BIC, HQ). For scenarios D and E, the simulation conditions are the number of items (p=10,20), sample size (n=500,1000), test statistics (${\text{GH}}_{T1},{\text{GH}}_{T2}$, ${\text{LR}}_{1}$, ${\text{LR}}_{2}$, ${\bar{X}}^{2}$, $M_{2}$) and information criteria (AIC, BIC, HQ). We have considered two nominal levels of α, α=.05,.01 and R=500 replications for all scenarios.

Preliminary results from Guastadisegni et al. (2023) indicate that the ${\text{GH}}_{T1}$ test performs well under scenarios A and C, particularly for large sample sizes, with reasonable rates of Type I error and power.

Additional results that include the bias for the parameter estimates of the model ${\text{SNP}}_{0}$, ${\text{SNP}}_{1}$ and ${\text{SNP}}_{2}$ have been reported in Section S2.1 of Data S1. When the mean of the true latent variable differs from 0, and the variance differs from 1, as in scenarios B, C, D and E, to compute the parameter bias, the true $\alpha_{00}$ and $\alpha_{01}$ parameters have been rescaled accordingly to formulas (S12) and (S13) in Section S1.3 of Data S1, replacing ${\dot{\alpha }}_{0j}$ with the true intercept, ${\dot{\alpha }}_{1j}$ with the true slope, $\tilde{E}(Z)$ and $\tilde{V}(Z)$ with the mean and the variance of the true latent variable. In this way, the parameter bias is due only to the misspecification of the shape of the latent variable and not to the misspecification of the moments. A similar procedure to compute parameter bias has been adopted by Irincheeva (2011).

### 6.2 Results

Table 1 reports the Type I error rates of the ${\text{GH}}_{T1}$, ${\text{LR}}_{1}$, ${\bar{X}}^{2}$ and $M_{2}$ for scenario A.

**TABLE 1:** Scenario A: Type I error rates of the ${\text{GH}}_{T1}$, ${\text{LR}}_{1}$, ${\bar{X}}^{2}$ and $M_{2}$, p=10,20, n=500,1000,5000.

p	n	α=.05				α=.01			
		${\text{GH}}_{T1}$	$M_{2}$	${\text{LR}}_{1}$	${\bar{X}}^{2}$	${\text{GH}}_{T1}$	$M_{2}$	${\text{LR}}_{1}$	${\bar{X}}^{2}$
10	500	**.018**	.066	**0**	**.024**	.002	.012	0	.006
	1000	.044	.046	**.002**	.038	.006	.01	0	.014
	5000	.056	.04	.042	.052	.02	.002	0	.012
20	500	**.026**	.036	**.006**	.056	.008	0	0	.01
	1000	.042	.054	.054	.06	.008	.006	0	.012
	5000	.042	.05	**.264**	.052	.01	.016	**.094**	.018

*Note*: Values in boldface indicate that the nominal level α is not included in their confidence interval.

The ${\text{GH}}_{T1}$ and ${\bar{X}}^{2}$ tests exhibit good Type I error rates under most conditions. However, ${\text{GH}}_{T1}$ is more conservative than expected, but only when the sample size is small, and there are 10 or 20 items with α=.05. The ${\bar{X}}^{2}$ test has lower than expected Type I error rates with a small sample size, 10 items and α=.05. $M_{2}$ performs well regarding Type I error rates for both values of α, number of items and sample sizes. The ${\text{LR}}_{1}$ test performs the worst of the four tests. When there are 10 items and the sample size is 500 or 1000, or when there are 20 items, the sample size is 500, and α=.05 rejects more than it should. Moreover, for every level of α considered, it has seriously inflated Type I error rates with 20 items and n=5000.

Table 2 shows the percentages of times AIC, BIC and HQ select ${\text{SNP}}_{0}$ over ${\text{SNP}}_{1}$ for scenario A.

**TABLE 2:** Scenario A: Percentages of times AIC, BIC and HQ select ${\text{SNP}}_{0}$ over ${\text{SNP}}_{1}$, p=10,20, n=500,1000,5000.

p	n	AIC (%)	BIC (%)	HQ (%)
10	500	97	100	99.8
	1000	93.2	100	98.8
	5000	90.6	100	96.6
20	500	87.8	99.8	99.2
	1000	78.4	100	94.6
	5000	54.4	97	78.4

Among the three information criteria considered, the AIC has the worst performance. This is particularly noticeable with 20 items and all sample sizes, where it selects the ${\text{SNP}}_{0}$ model from 54% to 88% of times. On the contrary, the BIC performs the best, selecting the ${\text{SNP}}_{0}$ model almost every time under all conditions. The HQ performs moderately well in selecting the ${\text{SNP}}_{0}$ model, with its performance lying between that of the AIC and BIC criteria.

Table 3 presents the empirical power of the ${\text{GH}}_{T1}$, ${\text{LR}}_{1}$, ${\bar{X}}^{2}$ and $M_{2}$ for scenarios B and C.

**TABLE 3:** Scenarios B and C: Empirical power of the ${\text{GH}}_{T1}$, ${\text{LR}}_{1}$, ${\bar{X}}^{2}$ and $M_{2}$, p=10,20, n=500,1000,5000.

SC	p	n	α=.05				α=.01			
			${\text{GH}}_{T1}$	$M_{2}$	${\text{LR}}_{1}$	${\bar{X}}^{2}$	${\text{GH}}_{T1}$	$M_{2}$	${\text{LR}}_{1}$	${\bar{X}}^{2}$
B	10	500	.612	.03	.572	.508	.536	.004	.462	.282
		1000	.868	.028	.792	.866	.796	.006	.558	.672
		5000	1	.052	.936	1	1	.014	.93	1
	20	500	.886	.022	.596	.728	.76	.006	.428	.472
		1000	.984	.036	.63	.984	.96	.002	.60	.928
		5000	.996	.034	.678	1	.996	.004	.666	1
C	10	500	.924	.01	.912	.992	.852	.002	.8	.976
		1000	.998	.006	.968	1	.994	0	.96	1
		5000	1	.028	.972	1	1	.004	.972	1
	20	500	.988	0	.782	1	.95	0	.72	1
		1000	.996	0	.822	1	.994	0	.756	1
		5000	.998	.004	.922	1	.998	0	.892	1

Regarding the four tests and both levels of α considered, it was found that ${\text{GH}}_{T1}$ consistently exhibits higher power than the other tests under scenario B for small sample sizes. Under scenario C, and generally as the sample size increases, ${\text{GH}}_{T1}$ and ${\bar{X}}^{2}$ tend to have very similar power, which is consistently higher than ${\text{LR}}_{1}$. Overall, the power of the ${\text{GH}}_{T1}$, ${\text{LR}}_{1}$ and ${\bar{X}}^{2}$ tests increases as both the sample size and the number of items increase, and as the true latent variable distribution significantly diverges from the normal distribution. On the contrary, the $M_{2}$ test has very little or no power to detect non‐normality of the latent variable distribution under both scenarios considered.

Table 4 shows the percentages of times AIC, BIC and HQ select ${\text{SNP}}_{1}$ over ${\text{SNP}}_{0}$ for scenarios B and C.

**TABLE 4:** Scenarios B and C: Percentages of times AIC, BIC and HQ select ${\text{SNP}}_{1}$ over ${\text{SNP}}_{0}$, p=10,20, n=500,1000,5000.

SC	p	n	AIC (%)	BIC (%)	HQ (%)
B	10	500	75.6	47.6	58.8
		1000	85.2	55.4	78.8
		5000	94	93	93.4
	20	500	66.2	47	60.2
		1000	65.2	60	63
		5000	70	65.4	67.6
C	10	500	95.6	80.6	91.8
		1000	97	95.6	96.8
		5000	97.6	97.2	97.2
	20	500	85.8	73	79.4
		1000	87.6	75.4	82
		5000	95	87.2	92

In all cases, AIC performs best when the sample size is 500 or 1000, with the highest percentage of selecting the ${\text{SNP}}_{1}$ model. On the contrary, BIC has the poorest performance with sample size of 500 or 1000, especially when it comes to selecting the ${\text{SNP}}_{1}$ model under scenario B and a sample size of 500, where it only selects it around 47% of the time. HQ performs better than BIC but not as well as AIC in selecting the ${\text{SNP}}_{1}$ model under all scenarios for small sample sizes.

Table 5 reports the empirical power of the ${\text{GH}}_{T1}$, ${\text{LR}}_{1}$, ${\text{GH}}_{T2}$, ${\text{LR}}_{2}$, $M_{2}$ and ${\bar{X}}^{2}$ for scenarios D and E.

**TABLE 5:** Scenarios D and E: Empirical power of the ${\text{GH}}_{T1}$, ${\text{LR}}_{1}$, ${\text{GH}}_{T2}$, ${\text{LR}}_{2}$, $M_{2}$ and ${\bar{X}}^{2}$ tests, p=10,20, n=500,1000.

SC	p	n	α=.05						α=.01					
			${\text{GH}}_{T1}$	${\text{LR}}_{1}$	${\text{GH}}_{T2}$	${\text{LR}}_{2}$	$M_{2}$	${\bar{X}}^{2}$	${\text{GH}}_{T1}$	${\text{LR}}_{1}$	${\text{GH}}_{T2}$	${\text{LR}}_{2}$	$M_{2}$	${\bar{X}}^{2}$
D	10	500	.554	.374	.492	.7	.024	.39	.416	.27	.338	.462	.004	.186
		1000	.764	.588	.722	.956	.028	.742	.636	.264	.608	.85	.008	.504
	20	500	.786	.358	.89	.972	.026	.69	.576	.196	.762	.876	.008	.484
		1000	.962	.38	.992	1	.02	.972	.924	.348	.972	1	.002	.898
E	10	500	.588	.43	.562	.818	.028	.548	.458	.334	.404	.62	.01	.282
		1000	.894	.736	.814	.98	.036	.844	.834	.448	.688	.92	.014	.688
	20	500	.874	.45	.916	.99	.022	.808	.71	.282	.836	.946	.006	.626
		1000	.986	.474	1	1	.026	.996	.948	.458	.998	1	.026	.996

Under scenarios D and E, ${\text{GH}}_{T1}$ and ${\text{GH}}_{T2}$ generally exhibit very similar power across all conditions. For small sample sizes, the power of these tests is consistently higher than that of ${\bar{X}}^{2}$. For large sample sizes, ${\text{GH}}_{T1}$, ${\text{GH}}_{T2}$ and ${\bar{X}}^{2}$ demonstrate similar power, and it increases as the skew‐normal distribution becomes more extreme. ${\text{LR}}_{1}$ has low power under all conditions, while ${\text{LR}}_{2}$ exhibits the highest power among all scenarios. Although increasing the degree of the polynomial can enhance the LR test performance, the ${\text{SNP}}_{2}$ method requires accurate initial values for the parameters to yield good results, making it challenging to use in practice. As observed in scenarios B and C, the $M_{2}$ test has very low or no power in detecting misspecifications of the latent variable distribution.

Table 6 shows the percentages of times AIC, BIC and HQ select ${\text{SNP}}_{1}$ over ${\text{SNP}}_{0}$ and ${\text{SNP}}_{2}$ over ${\text{SNP}}_{0}$ for scenarios D and E.

**TABLE 6:** Scenarios D and E: Percentages of times AIC, BIC and HQ select ${\text{SNP}}_{1}$ over ${\text{SNP}}_{0}$ and ${\text{SNP}}_{2}$ over ${\text{SNP}}_{0}$, p=10,20, n=500,1000.

SC	p	n	${\text{SNP}}_{1}$ over ${\text{SNP}}_{0}$			${\text{SNP}}_{2}$ over ${\text{SNP}}_{0}$		
			AIC (%)	BIC (%)	HQ (%)	AIC (%)	BIC (%)	HQ (%)
D	10	500	58.4	28.4	39	87	26.2	60.4
		1000	70.4	24.4	58.4	99	61.8	90
	20	500	44.8	22	36.6	98.8	75.4	94
		1000	41.4	34.6	38	100	98.2	100
E	10	500	64.6	34.4	44.2	91.6	42.2	72.6
		1000	81.2	44	73.2	99	76	95
	20	500	51.2	31	45.2	99.6	84.4	97.8
		1000	49.6	45.6	47.4	100	99.6	100

In both scenarios and with different sample sizes, none of the criteria were effective in choosing between ${\text{SNP}}_{1}$ and ${\text{SNP}}_{0}$. However, because the true latent variables are skewed and ${\text{SNP}}_{2}$ better approximates this type of distribution, all the information criteria performed better when selecting between ${\text{SNP}}_{0}$ and ${\text{SNP}}_{2}$. Among the criteria, AIC showed the best performance, while BIC performed the worst. Overall, based on the results in Tables 2, 4 and 6, it was not possible to determine which criterion performed the best under normality and non‐normality of the latent variable.

To assess the performance of the ${\text{GH}}_{T}$ test in the presence of misspecification of the IRF, an additional scenario, referred to as **F**, was examined. Details of the simulation conditions and results are in Section S2.3 of Data S1. The results indicate that ${\text{GH}}_{T1}$ has lower power than ${\bar{X}}^{2}$. This suggests that if the ${\text{GH}}_{T1}$ test rejects the null hypothesis, it is more likely due to misspecification in the latent variable distribution rather than in the IRF specification.

## 7 REAL DATA APPLICATIONS

### 7.1 Grade 12 science assessment test

The Grade 12 science assessment test (SAT12) dataset can be found in the TESTFACT manual (Wood et al., 2003) and is also included in the R package mirt (Chalmers, 2012). It comprises 32 binary‐scored items measuring chemistry, biology and physics knowledge. Previous analysis by Monroe (2021) revealed that the 2PL model provided a poor fit, as indicated by the significant values of $R_{B}$ and ${\bar{X}}^{2}$. To further analyse this dataset, the ${\text{SNP}}_{0}$ and ${\text{SNP}}_{1}$ models were fitted to the same dataset used by Monroe (2021), which included 572 complete cases.

Section S3.1 of Data S1 reports estimates for the ${\text{SNP}}_{0}$ and ${\text{SNP}}_{1}$ models. These estimates show some differences, but they are not too large. To choose between the classic 2PL model and a more complex one, we first assessed the fit of the ${\text{SNP}}_{0}$ model. As the data are sparse, with all 570 observed response patterns having expected frequencies <5, we examined residuals calculated from marginal frequencies. We have used the rule that residuals >4 indicate a bad fit of the correspondent pair or triplets of items (Bartholomew et al., 2011). Although the results are not presented in the tables, the ${\text{SNP}}_{0}$ model exhibits a poor fit for specific pairs and triplets of items. We computed information criteria to assess whether the ${\text{SNP}}_{1}$ model would better fit.

Table 7 reports the AIC, BIC and HQ criteria for the ${\text{SNP}}_{0}$ and ${\text{SNP}}_{1}$ models. Based on the information criteria selected, the ${\text{SNP}}_{1}$ model is preferred, indicating the non‐normality of the latent variable.

**TABLE 7:** SAT12: Information criteria for the ${\text{SNP}}_{0}$ and ${\text{SNP}}_{1}$ models.

	AIC	BIC	HQ
${\text{SNP}}_{0}$	18,239.49	18,517.84	18,348.08
${\text{SNP}}_{1}$	18,199.7	18,482.39	18,309.98

Table 8 reports the value of the $M_{2}$, ${\text{LR}}_{1}$, ${\text{GH}}_{T1}$, ${\bar{X}}^{2}$ and $R_{B}$, the degrees of freedom (dof) and the associated p‐values.

**TABLE 8:** SAT 12 data: $M_{2}$, ${\text{LR}}_{1}$, ${\text{GH}}_{T1}$, ${\bar{X}}^{2}$ and $R_{B}$ test statistics and associated degrees of freedom and p‐values.

Test	Value	dof	p‐value
$M_{2}$	675.53	464	<.001
${\text{LR}}_{1}$	41.79	1	<.001
${\text{GH}}_{T1}$	40.775	6.80	<.001
${\bar{X}}^{2}$	172.8	30	<.001
$R_{B}$	7.59	–	<.001/31

The values of $R_{B}$ and ${\bar{X}}^{2}$ were obtained from Monroe (2021). The $R_{B}$ indicates that the latent variable is not normally distributed. The test $M_{2}$ rejects the null hypothesis that ${\text{SNP}}_{0}$ fits the data well. The ${\text{LR}}_{1}$ suggests a better fit for the ${\text{SNP}}_{1}$ model. Both ${\text{GH}}_{T1}$ and ${\bar{X}}^{2}$ reject the null hypothesis that the latent variable is normally distributed. As some of the parameter estimates are more extreme than those considered in the simulation study in Section 6, the reliability of the test results was evaluated through a simulation study that mimics the real dataset, as explained in Section S3.1 of Data S1. The findings indicate that all tests perform well in terms of Type I error rates and power, except for the $M_{2}$ test, which shows very low or no power in identifying non‐normality in the latent variable distributions.

This data analysis did not consider the ${\text{GH}}_{T2}$ and ${\text{LR}}_{2}$ tests as we already rejected the null hypothesis when considering only L=1.

### 7.2 The NLSF dataset

This study is based on data collected from the National Longitudinal Survey of Freshmen (NLSF), which is a project funded by the Mellon Foundation and the Atlantic Philanthropies (available at http://oprdata.princeton.edu/archive/restricted), designed by Douglas S. Massey and Camille Z. Charles. The NLSF aims to collect data to explain the underachievement of minority groups in higher education. The data were collected from 1999 to 2003 in four waves to capture emergent psychological processes, measuring the degree of social integration and intellectual engagement. The survey included equal‐sized samples of first‐year white, black, Asian and Latino students entering selective colleges and universities. For this study, we have analysed only a part of the questionnaire that refers to 1999. Specifically, we have selected 21 binary items, whose descriptions are reported in Table 9. The first 9 items measure violence in the neighbourhood, while the remaining 12 measure violence in the school.

**TABLE 9:** NLSF data: Item description.

Item	Question
1	In your neighbourhood, before you were 10
	do you remember seeing homeless people on the street?
2	Prostitutes on street?
3	Gang members hanging out on the street?
4	Drug paraphernalia on the street?
5	People selling illegal drugs in public?
6	People using illegal drugs in public?
7	People drinking or drunk in public?
8	Physical violence in public?
9	Hearing the sound of gunshots?
10	In your grade school, did you see students fighting?
11	Students smoking?
12	Students cutting class?
13	Students cutting school?
14	Students verbally abusing teachers?
15	Did you see physical violence directed at teachers by students?
16	Vandalism of school or personal property?
17	Theft of school or personal property?
18	Students consuming alcohol?
19	Students taking illegal drugs?
20	Students carrying knives as weapons?
21	Students with guns?

The initial set of observations contained 3924 responses, with possible answers being ‘no’, ‘yes’, ‘don't know’ and ‘refused’. Responses including ‘don't know’ or ‘refused’ were excluded from the analysis. In addition, ‘no’ responses were coded as 0 and ‘yes’ responses as 1. The final dataset used for analysis contained 3860 responses.

A subset of 400 observations, along with the items listed in Table 9, was analysed by Cagnone and Viroli (2012), who fitted a latent trait model to the data, with latent variables distributed as a finite mixture of multivariate Gaussians, to achieve both dimension reduction and clustering simultaneously. Cagnone and Viroli (2012) found a good fit for the model with 2 factors distributed as a mixture of Gaussians, with the first 9 items listed in Table 9 loading highly on one factor, and the remaining 12 on the other factor.

In this work, we analyse the two item batteries separately. The results are reported in the following subsections.

#### 7.2.1 American students exposure to neighbourhood violence

This section presents the analysis results of the nine items that measure neighbourhood violence.

Despite the ${\text{SNP}}_{0}$ and ${\text{SNP}}_{1}$ methods being on the same scale, we observe different parameter estimates with very extreme values, as reported in Section S3.2.1 of Data S1.

We evaluated the fit of the ${\text{SNP}}_{0}$ model, considering the data's sparsity.

Specifically, 174 of the 231 observed response patterns have expected frequencies of <5. Residuals calculated from marginal frequencies were examined, revealing that the ${\text{SNP}}_{0}$ model does not fit well for some pairs and triplets of items.

Table 10 reports the values of the AIC, BIC and HQ criteria for the ${\text{SNP}}_{0}$ and ${\text{SNP}}_{1}$ models.

**TABLE 10:** NLSF data, nine items: Information criteria for the ${\text{SNP}}_{0}$ and ${\text{SNP}}_{1}$ models.

	AIC	BIC	HQ
${\text{SNP}}_{0}$	21,309.32	21,422.12	21,349.36
${\text{SNP}}_{1}$	21,303.67	21,422.73	21,345.93

The information criteria give conflicting results. Indeed, AIC and HQ select the ${\text{SNP}}_{1}$ model, while BIC the ${\text{SNP}}_{0}$ model.

Table 11 reports the value of the $M_{2}$, ${\text{LR}}_{1}$, ${\text{GH}}_{T1}$ and ${\bar{X}}^{2}$ test statistics, the degrees of freedom (dof) and the associated p‐values.

**TABLE 11:** NLSF data, nine items: $M_{2}$, ${\text{LR}}_{1}$ ${\text{GH}}_{T1}$, ${\bar{X}}^{2}$ test statistics and associated degrees of freedom and p‐values.

Test	Value	dof	p‐value
$M_{2}$	182.33	27	0
${\text{LR}}_{1}$	7.66	1	.006
${\text{GH}}_{T1}$	222.92	2.65	0
${\bar{X}}^{2}$	11.8	7	.106

The test results show contradictions. Specifically, the $M_{2}$ test indicates that the ${\text{SNP}}_{0}$ model does not fit the data well. For the ${\text{LR}}_{1}$ and ${\text{GH}}_{T1}$ tests, the null hypothesis – that the latent variable is normally distributed – is rejected. In contrast, the ${\bar{X}}^{2}$ test does not reject the null hypothesis of normality for the latent variable. To further investigate these contradictory results, a simulation study was conducted with nine items. The study used the ${\text{SNP}}_{0}$ estimates (intercepts and slopes) as the true values for generating data. These estimates deviate significantly from the true parameter values used in the simulation presented in Section 7. The simulation results, presented in Section S3.2.1 of Data S1, indicate that all tests, except for ${\text{LR}}_{1}$, perform well regarding Type I error rates and when the latent variable is generated from a mixture of normals. Limitations arise under skew‐normal scenarios. Specifically, the ${\bar{X}}^{2}$ and $M_{2}$ tests exhibit almost no power. While the ${\text{GH}}_{T1}$ test shows slightly better performance than ${\bar{X}}^{2}$, its power remains low. Moreover, the ${\text{SNP}}_{1}$ method encountered convergence issues in nearly 20% of the replications. The results of the ${\text{LR}}_{1}$ test are unreliable due to significantly inflated false positive rates. Upon examining the results in Table 11 and those in Data S1, we conjecture that the situation observed in the real data is similar to scenarios D and E, where the ${\text{GH}}_{T1}$ test demonstrates somewhat higher power than the ${\bar{X}}^{2}$ test. Nevertheless, we cannot trust the test results as the overall power is very low. Additionally, this dataset presents a specific challenge not encountered in the other data analysed or in the other simulation studies presented in the paper and Data S1: approximately 33% of the loadings for the ${\text{SNP}}_{0}$ model, which are used as data‐generating values in the simulation study, are large, exceeding a value of 3. This factor could contribute to instability in the results and impact the estimation process of the ${\text{SNP}}_{1}$ model. While the application results remain unresolved, they could serve as a starting point for further investigation, as discussed in Section 8.4.

#### 7.2.2 American students exposure to school violence

This section presents the analysis results on the 12 items that measure school violence. Also, in this battery of items, it has been observed that the parameter estimates reported in Section S3.2.2 of Data S1 are different despite the methods being on the same scale, and they exhibit extreme values. To choose the best model for the data, we first evaluated the fit of the ${\text{SNP}}_{0}$ model.

The data are sparse, with 227 out of the 302 observed response patterns having expected frequencies <5. Inspection of the bivariate and trivariate residuals showed that the ${\text{SNP}}_{0}$ model does not fit well.

Table 12 reports the values of the AIC, BIC and HQ criteria for the ${\text{SNP}}_{0}$ and ${\text{SNP}}_{1}$ models.

**TABLE 12:** NLSF data, 12 items: Information criteria for the ${\text{SNP}}_{0}$ and ${\text{SNP}}_{1}$ models.

	AIC	BIC	HQ
${\text{SNP}}_{0}$	30,322.3	30,472.5	30,375.64
${\text{SNP}}_{1}$	30,219.13	30,375.59	30,274.69

All three information criteria indicate that the ${\text{SNP}}_{1}$ model fits better than the ${\text{SNP}}_{0}$ model.

Table 13 reports the value of the $M_{2}$, ${\text{LR}}_{1}$, ${\text{GH}}_{T1}$ and ${\bar{X}}^{2}$ test statistics, the degrees of freedom (dof) and their associated p‐values.

**TABLE 13:** NLSF data, 12 items: $M_{2}$, ${\text{LR}}_{1}$, ${\text{GH}}_{T1}$ and ${\bar{X}}^{2}$ and associated degrees of freedom and p‐values.

Test	Value	dof	p‐value
$M_{2}$	979.39	54	<.001
${\text{LR}}_{1}$	105.17	1	<.001
${\text{GH}}_{T1}$	127.69	2.53	<.001
${\bar{X}}^{2}$	68.4	10	<.001

Based on the $M_{2}$ test results, the ${\text{SNP}}_{0}$ model does not fit the data well. However, it is essential to note that the $M_{2}$ test does not identify the specific reason for this poor fit. On the contrary, the ${\text{LR}}_{1}$, ${\text{GH}}_{T1}$ and ${\bar{X}}^{2}$ tests reject the null hypothesis that the latent variable is normally distributed. For this particular set of items, the test statistics and information criteria yield consistent results. This is evident from an additional simulation study we conducted, which mimics the parameter estimates from the ${\text{SNP}}_{0}$ model fitted to the 12 items. The results can be found in Section S3.2.2 of Data S1. In summary, the performance of the test statistics varies significantly based on the scenario being considered. None of the test statistics exhibit inflated false positive rates, and all tests demonstrate high power when the latent variable is generated from an extreme mixture of normals, including the $M_{2}$ test. The power is notably low only when the latent variable is generated from a skew‐normal distribution, irrespective of the level of skewness.

## 8 DISCUSSION

In the following subsections, we provide a summary of the work done in this paper, present the main findings with a focus on comparisons between the proposed tests and others, discuss the limitations of the ${\text{GH}}_{T}$ test and outline directions for future work.

### 8.1 Summary of work

We have expanded the utilization of the GH test to detect the non‐normality of the latent variable distribution in a unidimensional IRT model for binary data. The GH test has been derived through the comparison of the PL estimator of the 2PL model for binary data with the ML estimator of the SNP‐IRT model, which allows for a more flexible shape of the latent variable distribution. We have used a simpler version of the GH test, referred to as the ${\text{GH}}_{T}$ test, which does not require the inversion of the covariance matrix. Its approximated distribution has been derived using the moment matching method. Two versions of ${\text{GH}}_{T}$ have been considered. The first version is the ${\text{GH}}_{T1}$ test, based on the ${\text{SNP}}_{1}$. Through the simulation study in Section 6, as well as through three real data applications and the simulation studies based on these real applications reported in Data S1, we have compared the performance of the ${\text{GH}}_{T1}$ test with the $M_{2}$, ${\text{LR}}_{1}$ and ${\bar{X}}^{2}$ test statistics and computed three information criteria, such as AIC, BIC and HQ. We also evaluated the sensitivity of the tests mentioned above to misspecifications of the item response function, with results reported in Data S1. As the SNP density when L=1 can only capture bimodal and slightly skew‐normal distributions, we have used the SNP density with L=2 because this parameterization enables the representation of highly skew‐normal distributions for specific combinations of parameter values. Therefore, we employed the second version of ${\text{GH}}_{T}$ based on the ${\text{SNP}}_{0}$ model, namely ${\text{GH}}_{T2}$, and the ${\text{LR}}_{2}$ test only in the context of skew‐normal scenarios in the main simulation study of Section 6 and in the study based on the SAT12 dataset to evaluate the performance of these tests with many items.

### 8.2 Main findings

In the main simulation study discussed in Section 6, we used a standard range of values (without extreme values) to generate the intercepts and slopes of the items. The results showed that the ${\text{GH}}_{T1}$ test performs well in terms of Type I error rates under most conditions. Furthermore, it demonstrates the highest power for detecting non‐normality in the latent variable distribution across various scenarios, particularly with small sample sizes. Specifically, ${\text{GH}}_{T1}$ outperforms ${\bar{X}}^{2}$ in terms of power for small sample sizes in the majority of cases, while both tests exhibit similar power when sample sizes are large. The ${\text{GH}}_{T1}$ test consistently exhibits higher power than the ${\text{LR}}_{1}$ test, which can produce inflated or deflated Type I error rates under specific conditions. Additionally, Irincheeva (2011) noted some limitations in using the LR test within the framework of SNP models. Furthermore, the ${\text{GH}}_{T1}$ test outperforms the $M_{2}$ test in terms of power. While the $M_{2}$ performs well regarding Type I error rates, it shows very low or no power to detect non‐normality in the latent variable distribution. The only exception to this occurs in extreme mixture of normal distributions, as shown in simulation studies utilizing the NLSF dataset (with 9 and 12 items) reported in Data S1. In this scenario, the $M_{2}$ test shows high power to detect this misspecification. Similar findings regarding the low power of the $M_{2}$ test to identify non‐normality in the latent variable distribution have also been reported by Li and Cai (2018), Paek et al. (2019) and Monroe (2021). As pointed out by Li and Cai (2018), this phenomenon can be attributed to the fact that $M_{2}$ relies on the first‐ and second‐order margins of the underlying contingency table. To identify any misfit in the latent variable distribution, it may be necessary to incorporate information from higher‐order margins.

The ${\text{GH}}_{T1}$ test proves to be more effective than the information criteria in this context, as none of the criteria consistently outperforms the others. The simulations indicate that it is challenging to determine which information criterion performs best, whether the latent variable is normally or non‐normally distributed. The AIC often selects more complex models in the highest percentage of cases, regardless of the distribution of the latent variable. This tendency to favour models with more parameters can result in overfitting. On the contrary, the BIC performs best when the latent variable follows a normal distribution. This is because BIC applies a stronger penalty for the number of parameters compared to the AIC, making it more suitable for selecting parsimonious models. However, BIC performs poorly under non‐normal distributions for small sample sizes, as it tends to select models that are too simple and fail to capture the complexity of the data. The performance of the HQ criterion lies between that of AIC and BIC.

From the additional simulation studies reported in Data S1, we found that the ${\text{GH}}_{T1}$ test is less sensitive to misspecifications in the item response function compared to ${\bar{X}}^{2}$, particularly with a small number of items and small sample sizes. Therefore, if the ${\text{GH}}_{T1}$ test results in a rejection, it is more likely that the misspecification is related to the latent variable distribution rather than the item response functions.

The simulation study based on the SAT12 dataset demonstrated that the performance of both the ${\text{GH}}_{T1}$ and ${\text{GH}}_{T2}$ tests remains stable, even when the number of items increases and certain item parameter values become more extreme. Overall, the results for all tests and information criteria are consistent with those observed in the main simulation study discussed in Section 6. Finally, the ${\text{GH}}_{T1}$ and ${\text{GH}}_{T2}$ tests show similar performance under skew‐normal scenarios.

### 8.3 Limitations of the proposed test

We identified two limitations in the ${\text{GH}}_{T1}$ and ${\text{GH}}_{T2}$ tests discussed in this work mainly in the case of examples with large slopes. For the ${\text{GH}}_{T1}$ test, issues arose particularly during the simulation studies based on the NLSF dataset mentioned in Data S1. Specifically, when data were generated with extreme values for item slopes under skew‐normal scenarios, the power of the ${\text{GH}}_{T1}$ test was notably low. Additionally, in simulations involving nine items, the ${\text{SNP}}_{1}$ model, which the test relies on, encountered convergence problems. It is also important to note that neither ${\bar{X}}^{2}$ nor the information criteria performed well under the skew‐normal scenarios in the simulations based on the NLSF dataset, especially with nine items. Furthermore, both ${\text{LR}}_{1}$ and $M_{2}$ exhibited limitations in these particular simulation studies. The second limitation is on the ${\text{GH}}_{T2}$ test that requires estimation of the ${\text{SNP}}_{2}$ model, which poses a challenge as accurate initial values are necessary for the optimization process to attain a reliable approximation of the true latent variable and minimize bias in parameter estimates compared to ${\text{SNP}}_{0}$. Also the ${\text{LR}}_{2}$ test, which shows high power under the skew‐normal scenarios in the simulation studies, is affected by the same problem. As a result, applying the ${\text{SNP}}_{2}$ model and the tests based on it in real data analysis becomes impractical due to these demanding requirements.

### 8.4 Future research

Further studies could address the issue of initial values in SNP estimation when L>1, enhancing its applicability in practical contexts. The performance of the GH test, when implemented with higher‐order polynomials, could also be assessed through simulations and real data analysis. Increasing the degree of the polynomial allows for greater flexibility in modelling the shape of the latent variable distribution, which can improve both information criteria and test performance.

The limitations identified from the NLSF data application, particularly concerning the nine items, may serve as a foundation for further investigation. The GH test could be applied using other estimation methods and models different from SNP to improve performance, especially in situations where slope parameter values are more extreme.

Additionally, evaluating the performance of the GH test in the IRT context could involve examining other types of model violations, such as local dependence or violations of the item characteristic function. In these cases, it is important to consider other estimation methods that provide consistent estimates under model misspecification for the application of the test.

## AUTHOR CONTRIBUTIONS

**Lucia Guastadisegni:** software; writing – original draft; methodology; data curation; visualization. **Silvia Cagnone:** methodology; writing – review and editing; formal analysis; supervision; conceptualization; data curation. **Irini Moustaki:** methodology; conceptualization; writing – review and editing; supervision; formal analysis; writing – original draft; validation. **Vassilis Vasdekis:** conceptualization; methodology; writing – review and editing; supervision; formal analysis.

## CONFLICT OF INTEREST STATEMENT

The authors declare no potential conflicts of interest with respect to the research, authorship and/orpublication of this article.

## Supporting information


Data S1

## Data Availability

The data NLSF that support the findings of this study are openly available in the Data Archive at the Office of Population Research, Princeton University at http://oprdata.princeton.edu/archive/restricted. The data SAT12 that support the findings of this study are openly available in R.
