# Supplementary material for: The generalized Hausman test for detecting non‐normality in the latent variable distribution of the two‐parameter IRT model
Source: Br J Math Stat Psychol. 2024 Dec 26;78(3):734–56. doi: 10.1111/bmsp.12379 (PMC12516116; doi:10.1111/bmsp.12379)
Supplement: Supplementary file 1 — Data S1 [file BMSP-78-734-s001.pdf]

Supplementary Material for: The generalized Hausman test for detecting non-normality in the latent variable distribution of the two-parameter IRT model

S1 Additional details about the SNP-IRT model

S1.1 SNP parameterization

The parameterization of the polynomial coefficients considered was initially proposed by Zhang and Davidian (2001) and imposed

$$1 = \int_R P_L^2(z)\phi(z)dz = E\{P_L^2(w)\} = \mathbf{a}' E(\tilde{\boldsymbol{w}}\tilde{\boldsymbol{w}}')\mathbf{a} = \mathbf{a}' A\mathbf{a} \qquad \text{(S1)}$$

with $w \sim N(0,1)$, $P_L(w) = \mathbf{a}'\tilde{\boldsymbol{w}}$, and $\tilde{\boldsymbol{w}}' = (1, w, w^2, ..., w^L)$. The matrix A is positive definite by definition and $A = B'B$, where B is a positive definite matrix.

If $\mathbf{c} = B\mathbf{a}$, equation (S1) becomes $\mathbf{c}'\mathbf{c} = 1$ and $\mathbf{c}' = (c_1, ..., c_{L+1})$. The elements of $\mathbf{c}$ can be represented using a polar coordinate transformation as $c_1 = \sin\varphi_1$, $c_2 = \cos\varphi_1 \sin\varphi_2, \ldots, c_L = \cos\varphi_1 \ldots cos\varphi_{L-1} \sin\varphi_L$, $c_{L+1} = \cos\varphi_1 \cos\varphi_2 \ldots \cos\varphi_{L-1} \cos\varphi_L$, with angles $-\pi/2 < \varphi_l \leq \pi/2$, $l = 1, ..., L$. The density of the latent variable in equation (2) of Section 2 can be expressed as

$$h(z|\boldsymbol{\varphi}, L) = (\mathbf{a}'\tilde{\mathbf{z}})^2\phi(z), \qquad \text{(S2)}$$

where $\mathbf{a}$ can be obtained from $\mathbf{c}$ as $\mathbf{a} = B^{-1}\mathbf{c}$, $\tilde{\mathbf{z}}' = (1, z, z^2, ..., z^L)$ and $\boldsymbol{\varphi}' = (\varphi_1, ..., \varphi_L)$.

S1.2 The mean and variance of the SNP latent variable

To compute the final estimator $\hat{\boldsymbol{\alpha}}_0' = (\hat{\alpha}_{01}, \ldots, \hat{\alpha}_{0p})$ in formula (S12) and $\hat{\boldsymbol{\alpha}}_1' = (\hat{\alpha}_{11}, \ldots, \hat{\alpha}_{1p})$ in (S13) it is necessary to compute $\tilde{E}(Z)$ and $\tilde{V}(Z)$ for the latent variable with density given in equation (2), in Section 2. These quantities can be derived analytically.

From Zhang and Davidian (2001)

$$\tilde{E}(Z) = \boldsymbol{a}' M^* \boldsymbol{a}, \tag{S3}$$

where $\boldsymbol{a}$ includes the polynomial coefficients $a_0, \cdots, a_L$ of the SNP density, defined in Section S1.1. The element in the i-th row and j-th column of M^* is $E(z^{i+j-1})$ and $z \sim N(0,1)$. The matrix M^* includes the moments of a standard normal distribution.

When $L = 2$, $\boldsymbol{a}' = (a_0, a_1, a_2)'$ where $a_0 = sin\hat{\varphi}_1 - \frac{1}{\sqrt{2}} cos\hat{\varphi}_1 cos\hat{\varphi}_2$, $a_1 = cos\hat{\varphi}_1 sin\hat{\varphi}_2$, $a_2 = \frac{1}{\sqrt{2}} cos\hat{\varphi}_1 cos\hat{\varphi}_2$ and

$$M^* = E\begin{pmatrix} z & z^2 & z^3 \\ z^2 & z^3 & z^4 \\ z^3 & z^4 & z^5 \end{pmatrix} = \begin{pmatrix} 0 & 1 & 0 \\ 1 & 0 & 3 \\ 0 & 3 & 0 \end{pmatrix}. \tag{S4}$$

$$\begin{aligned}
\tilde{E}(Z) &= \begin{pmatrix} sin\hat{\varphi}_1 - \frac{1}{\sqrt{2}} cos\hat{\varphi}_1 cos\hat{\varphi}_2 & cos\hat{\varphi}_1 sin\hat{\varphi}_2 & \frac{1}{\sqrt{2}} cos\hat{\varphi}_1 cos\hat{\varphi}_2 \end{pmatrix} \begin{pmatrix} 0 & 1 & 0 \\ 1 & 0 & 3 \\ 0 & 3 & 0 \end{pmatrix} \begin{pmatrix} sin\hat{\varphi}_1 - \frac{1}{\sqrt{2}} cos\hat{\varphi}_1 cos\hat{\varphi}_2 \\ cos\hat{\varphi}_1 sin\hat{\varphi}_2 \\ \frac{1}{\sqrt{2}} cos\hat{\varphi}_1 cos\hat{\varphi}_2 \end{pmatrix} = \\
&= \begin{pmatrix} cos\hat{\varphi}_1 sin\hat{\varphi}_2 & sin\hat{\varphi}_1 + \frac{2}{\sqrt{2}} cos\hat{\varphi}_1 cos\hat{\varphi}_2 & 3cos\hat{\varphi}_1 sin\hat{\varphi}_2 \end{pmatrix} \begin{pmatrix} sin\hat{\varphi}_1 - \frac{1}{\sqrt{2}} cos\hat{\varphi}_1 cos\hat{\varphi}_2 \\ cos\hat{\varphi}_1 sin\hat{\varphi}_2 \\ \frac{1}{\sqrt{2}} cos\hat{\varphi}_1 cos\hat{\varphi}_2 \end{pmatrix} = \\
&= 2 sin\hat{\varphi}_1 cos\hat{\varphi}_1 sin\hat{\varphi}_2 + \frac{4}{\sqrt{2}} cos\hat{\varphi}_1{}^2 cos\hat{\varphi}_2 sin\hat{\varphi}_2
\end{aligned} \tag{S5}$$

To compute $\tilde{V}(Z)$ we need also $\tilde{E}(Z^2)$. It can be computed as $\mathbf{a}' M^{**} \mathbf{a}$, where the element in the i-th

row and j-th column of M^{**} is $E(z^{i+j})$, and $z \sim N(0,1)$ (Zhang and Davidian, 2001). When $L = 2$

$$M^{**} = E\begin{pmatrix} z^2 & z^3 & z^4 \\ z^3 & z^4 & z^5 \\ z^4 & z^5 & z^6 \end{pmatrix} = \begin{pmatrix} 1 & 0 & 3 \\ 0 & 3 & 0 \\ 3 & 0 & 15 \end{pmatrix} \tag{S6}$$

and

$$\tilde{E}(Z^2) = \begin{pmatrix} sin\hat{\varphi}1 - \frac{1}{\sqrt{2}}cos\hat{\varphi}_1 cos\hat{\varphi}_2 & cos\hat{\varphi}_1 sin\hat{\varphi}_2 & \frac{1}{\sqrt{2}}cos\hat{\varphi}_1 cos\hat{\varphi}_2 \end{pmatrix} \begin{pmatrix} 1 & 0 & 3 \\ 0 & 3 & 0 \\ 3 & 0 & 15 \end{pmatrix} \begin{pmatrix} sin\hat{\varphi}_1 - \frac{1}{\sqrt{2}}cos\hat{\varphi}_1 cos\hat{\varphi}_2 \\ cos\hat{\varphi}_1 sin\hat{\varphi}_2 \\ \frac{1}{\sqrt{2}}cos\hat{\varphi}_1 cos\hat{\varphi}_2 \end{pmatrix} =$$
$$= \begin{pmatrix} sin\hat{\varphi}_1 + \frac{2}{\sqrt{2}}cos\hat{\varphi}_1 cos\hat{\varphi}_2 & 3cos\hat{\varphi}_1 sin\hat{\varphi}_2 & 3sin\hat{\varphi}_1 + \frac{12}{\sqrt{2}}cos\hat{\varphi}_1 cos\hat{\varphi}_2 \end{pmatrix} \begin{pmatrix} sin\hat{\varphi}_1 - \frac{1}{\sqrt{2}}cos\hat{\varphi}_1 cos\hat{\varphi}_2 \\ cos\hat{\varphi}_1 sin\hat{\varphi}_2 \\ \frac{1}{\sqrt{2}}cos\hat{\varphi}_1 cos\hat{\varphi}_2 \end{pmatrix} =$$
$$= sin\hat{\varphi}_1{}^2 + \frac{4}{\sqrt{2}}cos\hat{\varphi}_1 sin\hat{\varphi}_1 cos\hat{\varphi}_2 + 3cos\hat{\varphi}_1{}^2 sin\hat{\varphi}_2{}^2 + 5cos\hat{\varphi}_1{}^2 cos\hat{\varphi}_2{}^2 \tag{S7}$$

The variance of the latent variable with an SNP density is computed as $\tilde{V}(Z) = \tilde{E}(Z^2) - \tilde{E}(Z)^2$, and we obtain

$$\begin{aligned} \tilde{V}(Z) = {} & sin\hat{\varphi}_1{}^2 + \frac{4}{\sqrt{2}}cos\hat{\varphi}_1 sin\hat{\varphi}1 cos\hat{\varphi}_2 + 3cos\hat{\varphi}_1{}^2 sin\hat{\varphi}_2{}^2 + 5cos\hat{\varphi}_1{}^2 cos\hat{\varphi}_2{}^2 - \\ & - (2sin\hat{\varphi}_1 cos\hat{\varphi}_1 sin\hat{\varphi}_2 + \frac{4}{\sqrt{2}}cos\hat{\varphi}_1{}^2 cos\hat{\varphi}_2 sin\hat{\varphi}_2)^2 \end{aligned} \tag{S8}$$

To get the mean and the variance of the latent variable for the SNP_1 model, $\hat{\varphi}_2$ should be set equal to $\frac{\pi}{2}$ in equations (S5) and (S8), thus giving, $\tilde{E}(Z) = 2sin\hat{\varphi}_1 cos\hat{\varphi}_1$ and $\tilde{V}(Z) = sin\hat{\varphi}_1{}^2 + 3cos\hat{\varphi}_1{}^2 - 4sin^2\hat{\varphi}_1 cos^2\hat{\varphi}_1$.

S1.3 Rescaling of parameter estimates

We provide the formulas for rescaling the parameter estimates of SNP to correspond to a latent variable distribution with mean 0 and variance 1, such as the 2PL model with a standard normal latent variable distribution.

$$\text{logit}(\pi_j(z)) = \mathring{\alpha}_{0j} + \mathring{\alpha}_{1j} z \qquad j = 1, ..., p \tag{S9}$$

and

$$z = \sqrt{\tilde{V}(Z)} z_1 + \tilde{E}(Z) \tag{S10}$$

where $\tilde{E}(Z)$ and $\tilde{V}(Z)$ are found given $\hat{\boldsymbol{\varphi}}$ and the SNP density of z and z_1 has the same distribution of z, but with mean 0 and variance 1.

If we substitute (S10) in (S9) we get

$$\text{logit}(\pi_j(z)) = \mathring{\alpha}_{0j} + \mathring{\alpha}_{1j} \sqrt{\tilde{V}(Z)} z_1 + \mathring{\alpha}_{1j} \tilde{E}(Z) \qquad j = 1, ..., p \tag{S11}$$

From equation (S11), we get the form of the final estimators, which corresponds to a latent variable with mean 0 and variance 1 (Irincheeva et al., 2012):

$$\hat{\alpha}_{0j} = \mathring{\alpha}_{0j} + \mathring{\alpha}_{1j} \tilde{E}(Z) \qquad j = 1, ..., p \tag{S12}$$

$$\hat{\alpha}_{1j} = \mathring{\alpha}_{1j} \sqrt{\tilde{V}(Z)} \qquad j = 1, ..., p, \tag{S13}$$

$\tilde{E}(Z)$ and $\tilde{V}(Z)$ can be computed analytically, given the values of $\hat{\boldsymbol{\varphi}}$, as shown in Section S1.2. Finally, $\hat{\boldsymbol{\theta}}^{(1)'} = (\hat{\boldsymbol{\alpha}}_0^{'}, \hat{\boldsymbol{\alpha}}_1^{'}, \hat{\boldsymbol{\varphi}}^{'})$. The final estimator $\hat{\boldsymbol{\theta}}$ corresponds to a latent variable with a mean of 0 and a variance of 1. This ensures that any differences in the parameter estimates are attributable to the shape of the latent variable rather than its location and scale.

S1.4 On the identification of the latent variable scale

To identify model parameters in equation (1) of Section 2, we need to show that model parameters are uniquely determined by data functions, for example, central moments (Lewbel, 2019). This Section aims to show that the angle φ_1 is at least a locally identified parameter when the latent variable z distribution follows the SNP_1 model.

Denote the linear predictor in equation (1) of Section 2 by $\eta_j = \alpha_{0j} + \alpha_{1j} z$, where z follows the SNP_1 model. It is easy to see that $E(\eta_j) = \alpha_{0j} + \alpha_{1j} E(z)$ and $V(\eta_j) = \alpha_{1j}^2 V(z)$. Suppose that our interest lies in the third central moment of η_j

$$E[(\eta_j - E(\eta_j))^3] = 2\alpha_{1j}^3 E(z)^3 - 3\alpha_{1j}^3 E(z)E(z^2) + \alpha_{1j}^3 E(z^3), \tag{S14}$$

where z follows an SNP_1 distribution. Therefore, we know from the formulae at the end of Section S1.2 that $E(z) = 2\sin\varphi_1\cos\varphi_1$ and $E(z^2) = \sin^2\varphi_1 + 3\cos^2\varphi_1$. The third moment of z can be found in a similar way to the first two moments as $E(z^3) = 6\sin\varphi_1\cos\varphi_1$.

Substituting to (S14), we get

$$\alpha_{1j} = \sqrt[3]{\frac{E[(\eta_j - E(\eta_j))^3]}{(16\sin^3\varphi_1\cos^3\varphi_1 - 3(2\sin\varphi_1\cos\varphi_1)(\sin^2\varphi_1 + 3\cos^2\varphi_1) + 6\sin\varphi_1\cos\varphi_1)}} \tag{S15}$$

Since, $Cov(\eta_j, \eta_k) = \alpha_{1j}\alpha_{1k}V(z)$, we get that

$$\frac{(E[(\eta_j - E(\eta_j))(\eta_k - E(\eta_k))])^3}{E[(\eta_j - E(\eta_j))^3]E[(\eta_k - E(\eta_k))^3]} = \frac{\frac{1}{8}(3 + 2\cos(2\varphi_1) + \cos(4\varphi_1))^3}{16\cos^6\varphi_1(1 + 2\cos 2\varphi_1)^2\sin^2\varphi_1} \tag{S16}$$

This means that the angle φ_1 can be determined by a function of the covariance between linear predictors for items j and k and their third central moments. The right hand of expression (S16) has five asymptotes to infinity when $\varphi_1 \to -\pi/2$ or $\varphi_1 \to \pi/2$, approximately to -1.047 or 1.047 and to 0.

A graph of this expression, denoted as $f(\varphi_1)$, is shown in Figure S1, from which we can understand that φ_1 is locally identified.

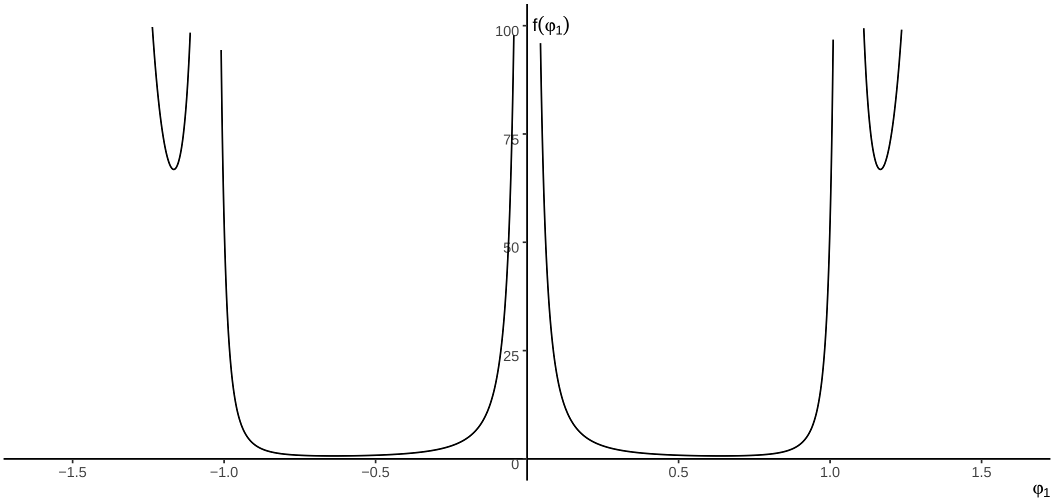

Figure S1: Graph of the right-hand side of expression (S16)

Once the local estimates of φ_1 are obtained, it is possible to estimate all model parameters, indicating that the model is locally identified without having to fix the latent variable to a mean of 0 and variance of 1 nor by constraining some of the factor loadings to specified values. This is why, in optimizing the log-likelihood function through the R function "nlminb," which minimizes the negative of the log-likelihood function, the algorithm always converged to the point that was at least a local minimum of the function. Indeed, we never encountered problems in the computation of the Hessian matrix, which was always positive definite, and invertible.

S2 Additional simulation results

S2.1 Parameter bias for the SNP_0, SNP_1 and SNP_2 models

In this Section, we report the bias observed in the ML estimates of the SNP_1 model and both ML and PL estimates of the SNP_0 under scenario C. Additionally, for scenario E, we report the bias of the ML

estimates of the SNP_2 model. The bias of each estimated parameter, $\hat{\theta}$, is given:

$$Bias_{\hat{\theta}} = \frac{1}{R}\sum_{l=1}^{R}(\hat{\theta}_l - \theta_0),$$

where θ_0 is the true parameter, $\hat{\theta}_l$ is the estimate of θ in the l-th replication, R replications equal to 500.

Table S1 presents the bias of the parameter estimates for the SNP_1 and SNP_0 models under scenario C, $p = 10$, $n = 5000$.

Table S1: Bias of the parameter estimates for the SNP_1 and SNP_0 models under scenario C, $p = 10$, $n = 5000$

θ	SNP_1(ML)	SNP_0(ML)	SNP_0(PL)
α_{0_1}	0.04	0.45	0.71
α_{0_2}	0.01	0.16	0.30
α_{0_3}	0.00	0.16	0.24
α_{0_4}	0.08	0.83	1.52
α_{0_5}	0.01	0.18	0.28
α_{0_6}	0.05	0.54	1.08
α_{0_7}	0.01	0.19	0.28
α_{0_8}	0.04	0.55	0.87
α_{0_9}	0.00	0.10	0.18
$\alpha_{0_{10}}$	0.00	0.03	0.06
α_{1_1}	0.05	0.51	0.69
α_{1_2}	0.01	0.28	0.41
α_{1_3}	0.01	0.14	0.17
α_{1_4}	0.11	0.96	1.52
α_{1_5}	0.02	0.25	0.33
α_{1_6}	0.06	0.70	1.17
α_{1_7}	0.01	0.20	0.25
α_{1_8}	0.06	0.61	0.83
α_{1_9}	0.01	0.19	0.28
$\alpha_{1_{10}}$	0.00	0.07	0.11

Table S2 presents the bias of the parameter estimates for the SNP_2, SNP_1 and SNP_0 models under scenario E, $p = 10$, $n = 1000$.

Table S2: Bias of the parameter estimates for the SNP_2, SNP_1 and SNP_0 models under scenario E, $p = 10$, $n = 1000$

θ	SNP_2(ML)	SNP_1(ML)	SNP_0(ML)	SNP_0(PL)
α_{0_1}	-0.01	-0.06	-0.12	-0.17
α_{0_2}	0.00	-0.02	-0.02	-0.03
α_{0_3}	0.00	-0.04	-0.07	-0.10
α_{0_4}	-0.01	-0.08	-0.15	-0.20
α_{0_5}	0.00	-0.03	-0.04	-0.06
α_{0_6}	0.00	-0.04	-0.07	-0.09
α_{0_7}	-0.01	-0.03	-0.07	-0.10
α_{0_8}	0.00	-0.07	-0.14	-0.20
α_{0_9}	0.00	-0.01	-0.02	-0.02
$\alpha_{0_{10}}$	0.00	-0.01	0.00	-0.01
α_{1_1}	0.00	0.04	0.14	0.20
α_{1_2}	0.00	-0.01	-0.01	0.00
α_{1_3}	0.00	0.05	0.11	0.17
α_{1_4}	0.01	0.05	0.13	0.18
α_{1_5}	-0.01	0.02	0.04	0.06
α_{1_6}	-0.01	0.02	-0.01	-0.01
α_{1_7}	0.01	0.02	0.11	0.15
α_{1_8}	-0.01	0.06	0.15	0.24
α_{1_9}	-0.01	0.00	-0.01	0.00
$\alpha_{1_{10}}$	0.00	0.00	0.00	0.01

S2.2 Graphs of SNP densities

This Section shows some graphs that display the actual non-normal latent variable densities used in the simulations. We also present the SNP densities for scenarios C and E, where the φ_1 and φ_2 parameters in the SNP densities correspond to the median parameter estimates obtained across replications.

Figure S2 displays the true density of the latent variable and the estimated SNP density with $L = 1$ and $\hat{\varphi}_1 = 0.56$ for scenario C, $p = 10$, and $n = 5000$.

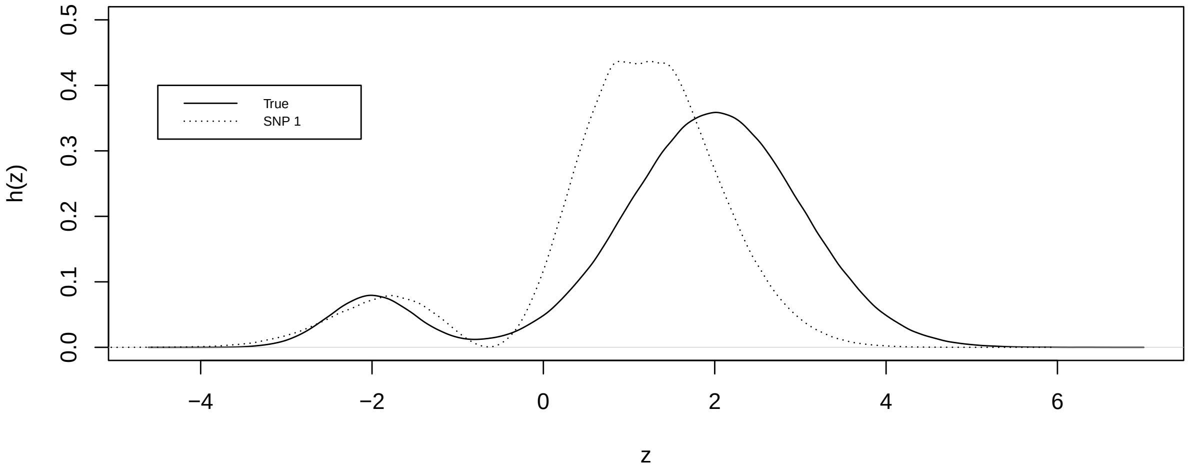

Figure S2: True density and estimated SNP density for scenario C

Figure S3 illustrates the actual density of the latent variable and the estimated SNP densities with $L = 1$ and $\hat{\varphi}_1 = -0.33$, and $L = 2$ with $\hat{\varphi}_1 = 1.10$ and $\hat{\varphi}_2 = -0.69$, for scenario E, $p = 10$ and $n = 1000$.

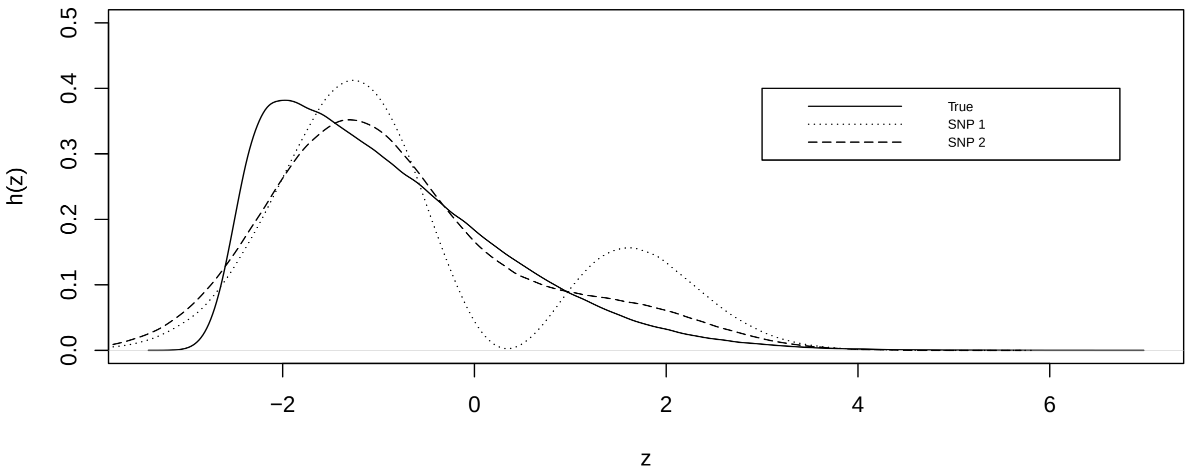

Figure S3: True density and estimated SNP densities for scenario E

S2.3 Sensitivity of the GH_T test statistic to misspecification of the item response function

An additional scenario called **F**, has been considered to study the behavior of the proposed test under item response function misspecification and correct specification of the latent variable distribution. The simulation conditions applied were the same as in Monroe (2021). Data was generated from a three-parameter logistic (3PL) model (Birnbaum, 1968), in which discrimination parameters α_{1j} were drawn from a log-normal distribution with a mean of 0 and a standard deviation of 0.25. Difficulty parameters, b_j, were drawn from a normal distribution with a mean of 0 and a standard deviation of 0.75. The intercept parameters were then calculated as $\alpha_{0j} = -b_j\alpha_{1j}$. Pseudo-guessing parameters were drawn from a beta distribution with parameters $\alpha = 8$ and $\beta = 32$. The latent variable has been generated from a standard normal. We have conducted simulations to test different conditions, including scenarios with 10 and 20 items, sample sizes of 500 and 1000, and 30 items with a sample size of 500. We analyzed all four test statistics, namely GH_{T1}, M_2, LR_1, and $\bar{X}^2$. The two-parameter logistic model was fitted to the

generated data. We used SNP_0 to calculate M_2, LR_1, $\bar{X}^2$, and SNP_1 for the GH_{T1} test. Table S3 reports the empirical power of the GH_{T1}, LR_1, GH_{T2}, LR_2, M_2 and $\bar{X}^2$ tests for scenarios F.

Table S3: Scenario F: Power of the GH_{T1}, M_2, LR_1 and $\bar{X}^2$ tests, $p = 10, 20, 30$, $n = 500, 1000$

		$\alpha = 0.05$				$\alpha = 0.01$			
p	n	GH_{T1}	M_2	LR_1	$\bar{X}^2$	GH_{T1}	M_2	LR_1	$\bar{X}^2$
10	500	0.01	0.034	0.432	0.196	0.006	0.004	0.16	0.064
	1000	0.05	0.066	0.616	0.274	0.012	0.01	0.4	0.128
20	500	0.246	0.032	0.812	0.548	0.088	0.002	0.694	0.37
	1000	0.698	0.05	0.966	0.878	0.544	0.006	0.858	0.758
30	500	0.306	0.038	0.844	0.63	0.138	0.01	0.712	0.478

Note 1: Values in boldface indicate that the nominal level α is not included in their confidence interval

The GH_{T1} test exhibits low to moderate levels of power in most cases examined. Its power increased as the number of items increased. It is less powerful than the LR_1 and $\bar{X}^2$ tests in all cases. M_2 was not found to detect this type of misspecification.

S3 Real data applications: additional results

S3.1 Grade 12 Science Assessment Test

Table S4 reports the ML estimates and standard errors based on the sandwich covariance matrix of the SNP_0 and SNP_1 models for the SAT12 data.

Table S4: SAT 12 data: SNP_0 and SNP_1 parameter estimates and standard errors in brackets

	Estimates			Estimates	
Parameter	SNP_0	SNP_1	Parameter	SNP_0	SNP_1
α_{0_1}	-1.02 (0.11)	-1.00 (0.10)	α_{1_1}	0.84 (0.12)	0.82 (0.11)
α_{0_2}	0.49 (0.11)	0.63 (0.13)	α_{1_2}	1.41 (0.17)	1.68 (0.25)
α_{0_3}	-1.10 (0.12)	-1.05 (0.11)	α_{1_3}	1.07 (0.14)	1.04 (0.13)
α_{0_4}	-0.52 (0.09)	-0.51 (0.09)	α_{1_4}	0.61 (0.11)	0.62 (0.11)
α_{0_5}	0.61 (0.10)	0.64 (0.11)	α_{1_5}	0.97 (0.14)	1.00 (0.20)
α_{0_6}	-2.01 (0.17)	-1.97 (0.15)	α_{1_6}	1.13 (0.17)	1.03 (0.13)
α_{0_7}	1.39 (0.13)	1.45 (0.15)	α_{1_7}	0.99 (0.16)	1.13 (0.24)
α_{0_8}	-1.44 (0.11)	-1.43 (0.11)	α_{1_8}	0.66 (0.13)	0.65 (0.11)
α_{0_9}	2.12 (0.14)	2.11 (0.14)	α_{1_9}	0.44 (0.16)	0.42 (0.19)
$\alpha_{0_{10}}$	-0.34 (0.10)	-0.31 (0.10)	$\alpha_{1_{10}}$	0.97 (0.13)	0.90 (0.15)
$\alpha_{0_{11}}$	5.16 (0.57)	5.40 (0.65)	$\alpha_{1_{11}}$	1.62 (0.41)	2.12 (0.57)
$\alpha_{0_{12}}$	-0.32 (0.08)	-0.32 (0.08)	$\alpha_{1_{12}}$	0.10 (0.09)	0.11 (0.09)
$\alpha_{0_{13}}$	0.85 (0.11)	0.90 (0.12)	$\alpha_{1_{13}}$	1.02 (0.14)	1.11 (0.20)
$\alpha_{0_{14}}$	1.19 (0.12)	1.26 (0.14)	$\alpha_{1_{14}}$	1.00 (0.14)	1.16 (0.22)
$\alpha_{0_{15}}$	1.98 (0.17)	2.09 (0.23)	$\alpha_{1_{15}}$	1.25 (0.21)	1.50 (0.33)
$\alpha_{0_{16}}$	-0.34 (0.09)	-0.32 (0.09)	$\alpha_{1_{16}}$	0.69 (0.11)	0.72 (0.11)
$\alpha_{0_{17}}$	4.11 (0.46)	4.25 (0.64)	$\alpha_{1_{17}}$	1.39 (0.41)	1.74 (0.72)
$\alpha_{0_{18}}$	-0.82 (0.13)	-0.67 (0.13)	$\alpha_{1_{18}}$	1.67 (0.19)	1.64 (0.24)
$\alpha_{0_{19}}$	0.22 (0.09)	0.25 (0.09)	$\alpha_{1_{19}}$	0.78 (0.11)	0.82 (0.14)
$\alpha_{0_{20}}$	2.69 (0.23)	2.92 (0.30)	$\alpha_{1_{20}}$	1.51 (0.24)	1.97 (0.37)
$\alpha_{0_{21}}$	2.56 (0.18)	2.58 (0.19)	$\alpha_{1_{21}}$	0.58 (0.21)	0.64 (0.27)
$\alpha_{0_{22}}$	3.43 (0.29)	3.67 (0.36)	$\alpha_{1_{22}}$	1.45 (0.27)	1.93 (0.40)
$\alpha_{0_{23}}$	-0.85 (0.09)	-0.84 (0.09)	$\alpha_{1_{23}}$	0.61 (0.11)	0.60 (0.10)
$\alpha_{0_{24}}$	1.31 (0.13)	1.43 (0.17)	$\alpha_{1_{24}}$	1.24 (0.17)	1.50 (0.28)
$\alpha_{0_{25}}$	-0.53 (0.09)	-0.51 (0.09)	$\alpha_{1_{25}}$	0.77 (0.12)	0.79 (0.12)
$\alpha_{0_{26}}$	-0.11 (0.11)	0.00 (0.12)	$\alpha_{1_{26}}$	1.44 (0.16)	1.62 (0.22)
$\alpha_{0_{27}}$	2.84 (0.28)	3.14 (0.39)	$\alpha_{1_{27}}$	1.90 (0.30)	2.50 (0.49)
$\alpha_{0_{28}}$	0.25 (0.10)	0.29 (0.10)	$\alpha_{1_{28}}$	1.07 (0.14)	1.06 (0.17)
$\alpha_{0_{29}}$	-0.72 (0.10)	-0.69 (0.10)	$\alpha_{1_{29}}$	0.86 (0.13)	0.87 (0.12)
$\alpha_{0_{30}}$	-0.20 (0.08)	-0.19 (0.08)	$\alpha_{1_{30}}$	0.31 (0.09)	0.29 (0.09)
$\alpha_{0_{31}}$	2.75 (0.28)	3.26 (0.44)	$\alpha_{1_{31}}$	2.18 (0.32)	3.10 (0.56)
$\alpha_{0_{32}}$	-1.62 (0.11)	-1.63 (0.11)	$\alpha_{1_{32}}$	0.07 (0.14)	0.13 (0.13)
			φ_1		-0.48 (0.10)

We conducted a simulation study to assess the performance of the proposed GH_T test statistic, along with GH_{T2} and LR_2 in a scenario involving a larger number of items ($p = 30$). The data were generated using parameter estimates from the SNP_0 for the first 30 items in Table S4 as the true item intercepts and slope values, based on a 2PL model. Some of these parameter estimates are more extreme than those

considered in the simulation study presented in Section 6. The latent variable was generated from the same scenarios outlined in Section 6. We used a sample size of 500 and considered 500 replications for each study condition. Table S5 gives the Type I error rates of the GH_{T1}, M_2, $\bar{X}^2$, LR_1 tests for scenario A where $p = 30$ and $n = 500$. Additionally, it reports the empirical power of the GH_{T1}, M_2, LR_1, $\bar{X}^2$ tests for scenarios B, C, D, and E along with the GH_{T2}, LR_2 tests for scenarios D and E, where $p = 30$ and $n = 500$.

Table S5: Type I error rates and power of the GH_{T1}, M_2, LR_1, GH_{T2} and LR_2 tests, $p = 30$, $n = 500$

	$\alpha = 0.05$						$\alpha = 0.01$					
SC	GH_{T1}	M_2	LR_1	$\bar{X}^2$	GH_{T2}	LR_2	GH_{T1}	M_2	LR_1	$\bar{X}^2$	GH_{T2}	LR_2
A	**0.012**	**0.08**	**0.008**	0.038	-	-	0.004	0.018	0	0.012	-	-
B	0.954	0.074	0.608	0.476	-	-	0.868	0.006	0.5	0.226	-	-
C	0.825	0.062	0.973	1	-	-	0.713	0.01	0.965	1	-	-
D	0.958	0.054	0.468	0.674	0.98	0.998	0.812	0.016	0.34	0.416	0.924	0.986
E	0.964	0.054	0.64	0.854	0.986	1	0.872	0.022	0.59	0.64	0.952	0.996

Note 1: Values in boldface indicate that the nominal level α is not included in their confidence interval

In terms of Type I error rates, only at the significance level $\alpha = 0.05$, GH_{T1} tends to under-reject the null hypothesis, as observed for 10 and 20 items in Section 6, and M_2 has an inflated Type I error rate. However, GH_{T1} consistently exhibits higher power than $\bar{X}^2$ under scenarios B, D, and E. Under scenario C, $\bar{X}^2$ has slightly higher power than GH_{T1}. GH_{T2} and LR_2 exhibit the highest power. In general, even though some of the intercepts and slopes used for generating the data have more extreme values than those considered in the simulation study in Section 6, the performance of the tests remains consistent.

Table S6 shows the percentage of times AIC, BIC, and HQ select SNP_0 instead of SNP_1 for scenario A, where $p = 30$, $n = 500$. It also shows the percentage of times AIC, BIC, and HQ select SNP_1 over SNP_0 for scenarios B, C, D, and E and SNP_2 over SNP_0 for scenarios D and E, where $p = 30$, $n = 500$.

Table S6: Percentages of times AIC, BIC, and HQ select SNP_0 over SNP_1 for scenario A, SNP_1 over SNP_0 for scenarios B, C, D, E, and SNP_2 over SNP_0 for scenarios D, E, $p = 30$, $n = 500$

	SNP_1 vs SNP_0			SNP_2 vs SNP_0		
SC	AIC	BIC	HQ	AIC	BIC	HQ
A	95.8%	100%	99%	-	-	-
B	68.4%	51.6%	61.6%	-	-	-
C	98.17%	96.54%	97.35%	-	-	-
D	56.8%	36.6%	47.6%	100%	95%	99.4%
E	68.4%	60.4%	64%	100%	98.8%	100%

Note: For scenario A we report SNP_0 over SNP_1

In scenario A, BIC and HQ are the most effective for choosing SNP_0 over SNP_1. However, when dealing with a mixture of normal distributions, AIC tends to prefer SNP_1 more often than SNP_0. HQ falls between BIC and AIC in terms of accuracy. Nevertheless, when the latent variable is generated from skew-normal distribution, these criteria are less reliable for confidently selecting SNP_1 over SNP_0. In scenarios D and E, the information criteria perform better when comparing SNP_0 and SNP_2. AIC performs the best, while BIC performs the worst. Furthermore, the performance of the information criteria aligns with what was observed in the simulation study of Section 6.

S3.2 The NLSF dataset

S3.2.1 American students exposure to neighbourhood violence

Table S7 presents the ML estimates and standard errors for the SNP_0 and SNP_1 models, based on the analysis of the 9 items measuring neighborhood violence in the NLSF dataset.

Table S7: NLSF data, 9 items: SNP_0 and SNP_1 parameter estimates and related standard errors

	Estimates	
Parameter	SNP_0	SNP_1
α_{0_1}	-1.85 (0.07)	-2.61 (0.13)
α_{0_2}	-5.21 (0.22)	-6.56 (0.34)
α_{0_3}	-3.43 (0.14)	-4.88 (0.25)
α_{0_4}	-5.05 (0.27)	-7.49 (0.52)
α_{0_5}	-6.85 (0.48)	-9.43 (0.68)
α_{0_6}	-5.75 (0.31)	-7.76 (0.46)
α_{0_7}	-1.61 (0.09)	-2.78 (0.17)
α_{0_8}	-1.99 (0.09)	-2.99 (0.16)
α_{0_9}	-2.76 (0.10)	-3.68 (0.18)
α_{1_1}	1.94 (0.09)	2.93 (0.18)
α_{1_2}	2.40 (0.15)	4.09 (0.28)
α_{1_3}	2.84 (0.15)	4.64 (0.26)
α_{1_4}	3.77 (0.23)	6.71 (0.47)
α_{1_5}	4.56 (0.37)	7.75 (0.57)
α_{1_6}	3.38 (0.22)	5.83 (0.38)
α_{1_7}	2.77 (0.15)	4.16 (0.26)
α_{1_8}	2.32 (0.11)	3.58 (0.21)
α_{1_9}	2.00 (0.10)	3.19 (0.19)
φ_1	-	0.23(0.04)

In light of the conflicting results from the test statistics discussed in Section 7.2.1, and the discrepancy between the parameter estimates in Table S7 and those used as the true values in the simulation study of Section 6, we performed a simulation study to assess the tests' under conditions similar to the NLSF application. We generated data from a 2PL model using the SNP_0 estimates from Table S7 for the true item intercepts and slope values. We replicated the scenarios considered in Section 6 to generate the latent variable. A sample size of 4000 was used, and we conducted 500 replications for each condition of the study. Additionally, we evaluated the performance of the information criteria.

Table S8 presents the Type I error rates for the GH_{T1}, M_2, $\bar{X}^2$, and LR_1 tests for scenario A. It also reports the empirical power of these tests for scenarios B, C, D, and E, with $p = 9$ and $n = 4000$.

Table S8: Type I error rates and power of the GH_{T1}, M_2, LR_1 and $\bar{X}^2$, $p = 9$, $n = 4000$ using as true parameter values the SNP_1 estimates

	$\alpha = 0.05$				$\alpha = 0.01$			
SC	GH_{T1}	M_2	LR_1	$\bar{X}^2$	GH_{T1}	M_2	LR_1	$\bar{X}^2$
A	**0.014**	0.04	**0.486**	0.044	0.004	0.016	**0.308**	0.012
B	0.362	0.124	0.646	0.726	0.297	0.038	0.544	0.458
C	0.952	0.708	0.788	1	0.858	0.47	0.716	1
D	0.223(19%)	0.03	0.611(19%)	0.078	0.048(19%)	0.01	0.514(19%)	0.014
E	0.246(20%)	0.034	0.623(20%)	0.08	0.038(20%)	0.006	0.493(20%)	0.01

Note 1: Values in boldface indicate that the nominal level α is not included in their confidence interval

Note 2: The percentage of times the SNP_1 model did not converge is reported in round brackets

In scenario A, the LR_1 test shows inflated Type I error rates, while the GH_{T1} test under-rejects the null hypothesis when $\alpha = 0.05$. However, the other tests perform well. The $\bar{X}^2$ test demonstrates the highest power in scenarios involving a mixture of normal distributions. The power of GH_{T1} is high, but only for scenario C. Under these true parameter values, M_2 reaches a power of around 0.7 and LR_1 around 0.8 in scenario C. When data are generated from skew-normal distributions, caution should be exercised when interpreting the results. Both M_2 and $\bar{X}^2$ tests show very low or no power in identifying this misspecification. Additionally, the SNP_1 model, on which the LR_1 and GH_{T1} tests are based, fails to converge in nearly 20% of the considered samples, resulting in unstable results.

Table S9 shows the percentage of times AIC, BIC, and HQ select SNP_0 over SNP_1 for scenario A. It also shows the percentage of times these criteria select SNP_1 over SNP_0 for scenarios B, C, D, and E, with $p = 9$ and $n = 4000$.

Table S9: Percentages of times AIC, BIC, and HQ select SNP_0 over SNP_1 for scenario A and SNP_1 over SNP_0 for scenarios B, C, D, E, $p = 9$, $n = 4000$

	SNP_0 vs SNP_1		
SC	AIC	BIC	HQ
A	39.4%	78%	53.4%
B	76.64%	48.07	63.49%
C	85.2%	67%	77.2%
D	67.9%(19%)	40%(19%)	60%(19%)
E	79%(20%)	48%(20%)	60%(20%)

Note 1: For scenario A we report SNP_0 over SNP_1

Note 2: The percentage of times the SNP_1 model did not converge is reported in round brackets

In scenario A, BIC performs the best, but it performs the worst in the other scenarios. AIC performs the best when the latent variable is generated from a mixture of normals. However, due to the same convergence issues of the SNP_1 model outlined in the computation of the GH_{T1} and LR_1 tests, the results of the information criteria are also unreliable under scenarios D and E.

S3.2.2 American students exposure to school violence

Table S10 presents the ML estimates and standard errors for the SNP_0 and SNP_1 models, based on the analysis of the 12 items measuring school violence in the NLSF dataset.

Table S10: NLSF data, 12 items: SNP_0 and SNP_1 parameter estimates and standard errors in brackets

	Estimates	
Parameter	SNP_0	SNP_1
α_{0_1}	0.92 (0.05)	0.81 (0.04)
α_{0_2}	-3.09 (0.12)	-3.96 (0.22)
α_{0_3}	-2.96 (0.20)	-5.96 (0.50)
α_{0_4}	-2.93 (0.23)	-6.46 (0.63)
α_{0_5}	-1.14 (0.05)	-1.33 (0.07)
α_{0_6}	-3.05 (0.09)	-3.33 (0.14)
α_{0_7}	-0.07 (0.05)	-0.27 (0.06)
α_{0_8}	-0.73 (0.05)	-0.91 (0.06)
α_{0_9}	-5.76 (0.34)	-6.85 (0.50)
$\alpha_{0_{10}}$	-7.58 (0.59)	-8.87 (0.78)
$\alpha_{0_{11}}$	-3.66 (0.14)	-4.29 (0.22)
$\alpha_{0_{12}}$	-6.09(0.42)	-6.65 (0.68)
α_{1_1}	1.51 (0.07)	1.19 (0.05)
α_{1_2}	1.79 (0.11)	2.92 (0.24)
α_{1_3}	2.33 (0.23)	5.90 (0.54)
α_{1_4}	2.52 (0.27)	6.75 (0.68)
α_{1_5}	1.59 (0.07)	1.76 (0.10)
α_{1_6}	1.45 (0.09)	1.91 (0.15)
α_{1_7}	2.01 (0.11)	1.90 (0.09)
α_{1_8}	1.49 (0.08)	1.67 (0.08)
α_{1_9}	1.86 (0.21)	3.21 (0.39)
$\alpha_{1_{10}}$	2.90 (0.32)	4.71 (0.56)
$\alpha_{1_{11}}$	1.84 (0.12)	2.73 (0.23)
$\alpha_{1_{12}}$	1.57 (0.26)	2.40 (0.56)
φ_1		0.20 (0.02)

We conducted a simulation study using true parameter values for generating the data the estimates shown in Table S10 under the same conditions as the set of nine items. The results are presented in the following tables.

Table S11 presents the Type I error rates for the GH_{T1}, M_2, $\bar{X}^2$, and LR_1 tests for scenario A and reports the empirical power of these tests for scenarios B, C, D, and E, with $p = 12$ and $n = 4000$.

Table S11: Type I error rates the GH_{T1}, M_2, LR_1 and $\bar{X}^2$, $p = 12$, $n = 4000$

	$\alpha = 0.05$				$\alpha = 0.01$			
SC	GH_{T1}	M_2	LR_1	$\bar{X}^2$	GH_{T1}	M_2	LR_1	$\bar{X}^2$
A	**0.03**	0.06	**0.01**	0.032	0.006	0.016	0	0.004
B	0.616	0.256	0.258	0.982	0.284	0.11	0.134	0.936
C	0.996	0.892	0.928	1	0.996	0.744	0.914	1
D	0.243	0.05	0.426	0.346	0.113	0.006	0.2	0.114
E	0.206	0.048	0.480	0.426	0.106	0.004	0.267	0.184

Note 1: Values in boldface indicate that the nominal level α is not included in their confidence interval

In scenario A, for $\alpha = 0.05$, the GH_{T1} and LR_1 tests slightly under-reject the null hypothesis, while the other tests perform well. When the latent variable is generated from a mixture of normals, GH_{T1} and $\bar{X}^2$ perform the best, with the latter showing higher power under scenario B. Only under scenario C, M_2 exhibits high power. When the latent variable is generated from skew-normal, all tests show very low or no power to detect this type of misspecification.

Table S12 shows the percentage of times AIC, BIC, and HQ choose SNP_0 over SNP_1 in scenario A, where $p = 12$, $n = 4000$. It also presents the percentage of times these criteria choose SNP_1 over SNP_0 for scenarios B, C, D, and E where $p = 12$, $n = 4000$.

Table S12: Percentages of times AIC, BIC, and HQ select SNP_0 over SNP_1 for scenario A, SNP_1 over SNP_0 for scenarios B, C, D, E, $p = 12$, $n = 4000$

	SNP_0 vs SNP_1		
SC	AIC	BIC	HQ
A	98.2%	100%	99%
B	43.6%	8.4%	23%
C	91.4%	90.8%	92.4%
D	63.47%	13.91%	37.39%
E	61.06%	21.37%	45.03%

Note: For scenario A we report SNP_0 over SNP_1

In scenario A, all information criteria perform well and correctly select the SNP_0 model in nearly all cases. However, in scenario B, when the latent variable is generated from a mixture of normals, the performance of the information criteria is poor. It improves as the mixture becomes more extreme. On the other hand, when the latent variable is generated from a skewed normal, only AIC selects SNP_1 in 61.06% of cases, while the other information criteria perform very poorly.

References

Birnbaum, A. L. (1968). Some latent trait models and their use in inferring an examinee's ability. In Lord, F. M. and Novick, M. R., editors, *Statistical Theories of Mental Test Scores*, pages 397–479. Addison-Wesley.

Irincheeva, I., Cantoni, E., and Genton, M. G. (2012). Generalized linear latent variable models with flexible distribution of latent variables. *Scandinavian Journal of Statistics*, 39(4):663–680.

Lewbel, A. (2019). The identification zoo: Meanings of identification in econometrics. *Journal of Economic Literature*, 57(4):835–903.

Monroe, S. (2021). Testing latent variable distribution fit in IRT using posterior residuals. *Journal of Educational and Behavioral Statistics*, 46(3):374–398.

Zhang, D. and Davidian, M. (2001). Linear mixed models with flexible distributions of random effects for longitudinal data. *Biometrics*, 57(3):795–802.
